# Drug-Resistant Tuberculosis Case-Finding Strategies: Scoping Review

**DOI:** 10.2196/46137

**Published:** 2024-06-26

**Authors:** Susanna S van Wyk, Marriott Nliwasa, Fang-Wen Lu, Chih-Chan Lan, James A Seddon, Graeme Hoddinott, Lario Viljoen, Gunar Günther, Nunurai Ruswa, N Sarita Shah, Mareli Claassens

**Affiliations:** 1 Centre for Evidence Based Health Care Division of Epidemiology and Biostatistics Department of Global Health Stellenbosch University Cape Town South Africa; 2 Helse Nord Tuberculosis Initiative Kamuzu University of Health Sciences Blantyre Malawi; 3 Institute of Epidemiology and Preventive Medicine College of Public Health National Taiwan University Taipei Taiwan; 4 Desmond Tutu TB Centre, Department of Paediatrics and Child Health Faculty of Medicine and Health Sciences Stellenbosch University Cape Town South Africa; 5 Department of Infectious Disease Imperial College London London United Kingdom; 6 School of Public Health Faculty of Medicine and Health University of Sydney Sydney Australia; 7 Department of Pulmonary Medicine and Allergology Inselspital Bern University Hospital Bern Switzerland; 8 Department of Human, Biological & Translational Medical Science School of Medicine University of Namibia Windhoek Namibia; 9 National TB and Leprosy Programme Ministry of Health and Social Services Windhoek Namibia; 10 Departments of Epidemiology and Global Health Rollins School of Public Health Emory University Atlanta, GA United States

**Keywords:** tuberculosis, drug-resistant tuberculosis, drug-resistant tuberculosis case finding, drug-resistant tuberculosis case detection, drug-resistant tuberculosis screening, drug-resistant tuberculosis contact investigation, scoping review, TB symptom, anti-tuberculosis drug, strategies, multidrug-resistant, systematic review, drug resistant, drug resistance, medication, tuberculosis, diagnosis, screening

## Abstract

**Background:**

Finding individuals with drug-resistant tuberculosis (DR-TB) is important to control the pandemic and improve patient clinical outcomes. To our knowledge, systematic reviews assessing the effectiveness, cost-effectiveness, acceptability, and feasibility of different DR-TB case-finding strategies to inform research, policy, and practice, have not been conducted and the scope of primary research is unknown.

**Objective:**

We therefore assessed the available literature on DR-TB case-finding strategies.

**Methods:**

We looked at systematic reviews, trials, qualitative studies, diagnostic test accuracy studies, and other primary research that sought to improve DR-TB case detection specifically. We excluded studies that included patients seeking care for tuberculosis (TB) symptoms, patients already diagnosed with TB, or were laboratory-based. We searched the academic databases of MEDLINE, Embase, The Cochrane Library, Africa-Wide Information, CINAHL (Cumulated Index to Nursing and Allied Health Literature), Epistemonikos, and PROSPERO (The International Prospective Register of Systematic Reviews) using no language or date restrictions. We screened titles, abstracts, and full-text articles in duplicate. Data extraction and analyses were carried out in Excel (Microsoft Corp).

**Results:**

We screened 3646 titles and abstracts and 236 full-text articles. We identified 6 systematic reviews and 61 primary studies. Five reviews described the yield of contact investigation and focused on household contacts, airline contacts, comparison between drug-susceptible tuberculosis and DR-TB contacts, and concordance of DR-TB profiles between index cases and contacts. One review compared universal versus selective drug resistance testing. Primary studies described (1) 34 contact investigations, (2) 17 outbreak investigations, (3) 3 airline contact investigations, (4) 5 epidemiological analyses, (5) 1 public-private partnership program, and (6) an e-registry program. Primary studies were all descriptive and included cross-sectional and retrospective reviews of program data. No trials were identified. Data extraction from contact investigations was difficult due to incomplete reporting of relevant information.

**Conclusions:**

Existing descriptive reviews can be updated, but there is a dearth of knowledge on the effectiveness, cost-effectiveness, acceptability, and feasibility of DR-TB case-finding strategies to inform policy and practice. There is also a need for standardization of terminology, design, and reporting of DR-TB case-finding studies.

## Introduction

With the emergence of *Mycobacterium tuberculosis* strains resistant to first-line antituberculosis drugs, strategies to control tuberculosis (TB) have become even more challenging [1]. It is estimated that almost half a million people developed rifampicin-resistant TB, of which 78% had multidrug-resistant tuberculosis (MDR-TB) in 2019 [2]. Although drug-resistant tuberculosis (DR-TB) is not as prevalent as drug-susceptible tuberculosis (DS-TB), it is more difficult to diagnose, treatment is longer and more toxic, outcomes are worse, and costs are higher.

Finding individuals with DR-TB and initiating treatment as early as possible is important to improve patient clinical outcomes and to break the chain of transmission to help control the pandemic. Despite new diagnostic technologies, only a third of the estimated number of people who developed DR-TB initiated treatment in 2020 [3].

TB can be detected after the patient presents passively to health services or follows one of several different screening pathways depending on the case-finding strategy of a TB program [4]. Pathways can also be enhanced through several activities such as health promotion in the community, improved access to TB diagnostic services, or training of health workers to identify presumptive TB at general health services. Multiple activities often result in complex interventions and heterogeneous trials that are difficult to meta-analyze in systematic reviews [5,6].

To our knowledge, systematic reviews assessing the effectiveness, cost-effectiveness, acceptability, and feasibility of different DR-TB case-finding strategies to inform research, policy, and practice, have not been conducted and it is unknown whether enough research exists to conduct such reviews. It is also unknown whether case-finding strategies are similar for DR-TB and DS-TB and whether we can draw on findings from DS-TB reviews to inform decisions on DR-TB case-finding strategies.

Scoping reviews are useful for scoping the literature and to clarify concepts [7,8]. We therefore conducted a scoping review to assess whether enough research exists for a systematic review, to identify priority questions for such a review, and to clarify which case-finding strategies exist for DR-TB specifically.

## Methods

### Reporting Guidelines and Protocol

The Arksey and O’Malley framework [9], Levac et al [10], and the Joanna Briggs Institute scoping review methodology [8] guided methods for this scoping review. The review is reported according to the PRISMA-ScR (Preferred Reporting Items for Systematic Reviews and Meta-Analyses extension for Scoping Reviews) [11]. See Multimedia Appendix 1 for the completed PRISMA-ScR checklist. The protocol for this review was published in *JMIR Research Protocols* [12].

### Defining the Research Question

The question for our review was, what literature is available on DR-TB case finding and which case-finding strategies are described? We looked at studies that had sought to improve DR-TB case detection.

### Eligibility Criteria

Textbox 1 lists the inclusion and exclusion criteria for participants, concept, outcome, context, and study design.

**Eligibility criteria.**

**Inclusion criteria**

- Participants
- Participate regardless of symptoms, for example, contacts, people living with HIV attending HIV care, whole communities
- Concept/Intervention
- Strategies aiming to improve or enhance participants’ pathways to drug-resistant tuberculosis (DR-TB) case detection specifically
- Outcome
- Patients diagnosed with tuberculosis (TB)
- Context
- Community
- Primary, secondary, or tertiary care
- Study design
- Primary studies
- Systematic reviews
- Qualitative studies, where the experiences of individuals who receive the intervention or those who provide the intervention are investigated
- Studies of diagnostic test accuracy if the study describes a DR-TB screening strategy
- Trials comparing different screening or diagnostic tools within a DR-TB case-finding intervention

**Exclusion criteria**

- Participants
- Patients with TB symptoms seeking care
- Patients diagnosed with TB
- Laboratory samples/isolates
- Concept/Intervention
- Intervention strategies aiming to improve TB case finding in general, even if they do report the yield of people with DR-TB
- Outcome
- No report of patients diagnosed with TB
- Context
- Laboratory based
- Study design
- Meta-reviews (review of reviews)
- Narrative reviews
- Editorials
- Opinion articles
- Meeting summaries
- Guidelines
- Prevalence surveys, except if the survey includes an intervention strategy to find individuals with DR-TB specifically
- Conference abstracts

### Identifying Relevant Studies

With assistance from an information specialist, we searched the academic databases of MEDLINE (PubMed), Embase (Ovid), The Cochrane Library, Africa-Wide Information (EBSCOhost), CINAHL (EBSCOhost), Epistemonikos, and PROSPERO (The International Prospective Register of Systematic Reviews) using no language or date restrictions. These searches were conducted on August 31, 2021, and after an initial peer review of this article, an updated search was conducted on January 11, 2024.

The search string included combinations of the following 3 domains, that are (1) terms related to “TB”; (2) terms related to “drug resistance”; and (3) terms related to “case finding,” “case detection,” “screening,” “contact investigation,” and “contact tracing.”

Search strategies from each electronic database are detailed per search date in Multimedia Appendix 2.

### Study Selection

We used Rayyan systematic review software [13] to screen titles, abstracts, and full-text articles. Decisions were blinded, except when reviewing conflicts. Reviewers screened abstracts in duplicate for inclusion. Conflicts were resolved through discussion. Full-text articles were also screened in duplicate. Disagreements were resolved through discussion to determine final inclusion.

### Charting the Data

We developed a data extraction form in Excel (Microsoft Corp). The data extraction form was applied to all primary research reports to collect standard information on each study. Textbox 2 lists the information that was collected.

One reviewer extracted data from included papers and a second reviewer (SSvW) checked the extracted data. Reviewers met regularly to determine whether their approach was consistent and in line with the research question.

**Information collected on each study.**

- Authors, journal, year of publication
- Aim or purpose of the research
- Study design
- Country
- Income
- Tuberculosis prevalence
- HIV prevalence
- Urban or rural setting
- Participants
- Age
- Sex
- HIV status
- Other reported risk factors
- Target group and how the group was identified if applicable
- Interventions
- All components (activities) of the intervention
- Types of providers
- Screening and diagnostic tools used
- Treatment support, including preventive therapy
- Outcomes assessed

### Collating, Summarizing, and Reporting the Results

We provide a narrative report with supporting tables to summarize the data. Table 1 contains definitions we used in charting, collating, summarizing, and reporting our results.

**Figure 1:**
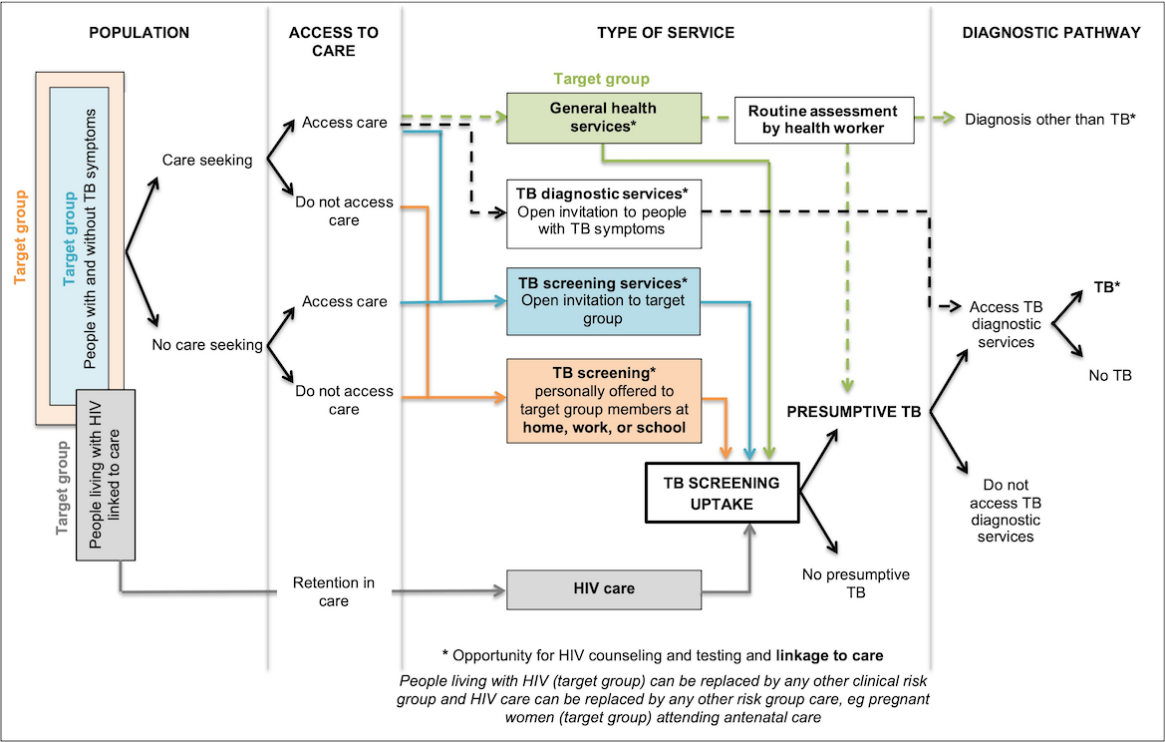
A systems-based logic model depicting types of services and associated pathways to tuberculosis (TB) case detection [16].

A systems-based logic model developed from a synthesis of DS-TB case-finding strategies (Figure 1) was used as a framework to describe different strategies and resulting pathways (care-seeking pathways or screening pathways).

Quality appraisal was not conducted, because this is a scoping review and our interest is in the existing evidence base, regardless of study design and quality.

**Table 1:** Definitions.

Terms	Definitions
DR-TB^a^	All types of DR-TB that include DR-TB with resistance to one first-line drug, MDR-TB^b^, XDR-TB^c^, and any other DR-TB reported by the authors.
Systematic screening for TB^d^ disease	“The systematic identification of people with suspected (presumptive) TB disease, in a predetermined target group, using tests, examinations, or other procedures that can be applied rapidly. Among those screened positive, the diagnosis needs to be established by one or several diagnostic tests and additional clinical assessments, which together have high accuracy.” [14]
A screening tool	Tests, examinations, or other procedures used for systematic screening for TB disease. Examples of TB screening tools include a structured symptom-based questionnaire, CXR^e^, or an algorithm [4]. Algorithms may include sequential or parallel tests. With sequential tests, only those who screen positive with the initial test receive a second test. With parallel tests, those who screen positive on any of the tests are regarded as screen positives.
A diagnostic tool	Tests, examinations, or other procedures used to establish a diagnosis of TB disease in people identified with presumptive TB. Examples of TB diagnostic tools include a clinical algorithm, sputum smear microscopy, Xpert MTB/RIF (Cepheid Inc), or culture [4].
TB symptom	Any TB symptom, for example, cough, fever, night sweats, weight loss, or combination of TB symptoms as defined by the study authors.
Care seeking	People seeking care for a perceived health problem.
TB care seeking	People seeking care for TB symptoms specifically.
A risk group	Any group of people in whom the prevalence or incidence of TB is significantly higher than in the general population. Examples of risk groups include a whole population within a geographical area or TB contacts [15].
A clinical risk group	Individuals diagnosed with a specific disease or condition that increases their risk for TB, for example, people living with HIV (PLHIV).
Presumptive TB	Presumptive TB is identified when a provider identifies a patient with suspected TB disease. In the context of screening, a person who screens positive is a patient with presumptive TB.
Passive case finding	Care-seeking pathway without TB screening, that is, the green and black dashed pathways in Figure 1 [16].
Passive case finding with an element of systematic screening or triage	TB screening at general health services, that is, the green pathway in Figure 1.
Enhanced case finding	TB health promotion with or without TB screening.
Active case finding	TB screening at TB screening services or at home, work, or school, that is, the blue and orange pathways in Figure 1. If the target group is TB contacts, this can also be referred to contact tracing or contact investigation.
Intensified case finding	TB screening of a clinical risk group, for example, people living with HIV (ie, the gray pathway in Figure 1).

^a^DR-TB: drug-resistant tuberculosis.

^b^MDR-TB: multidrug-resistant tuberculosis.

^c^XDR-TB: extensively drug-resistant tuberculosis.

^d^TB: tuberculosis.

^e^CXR: chest radiography.

## Results

### Overview of the Available Literature

**Figure 2:**
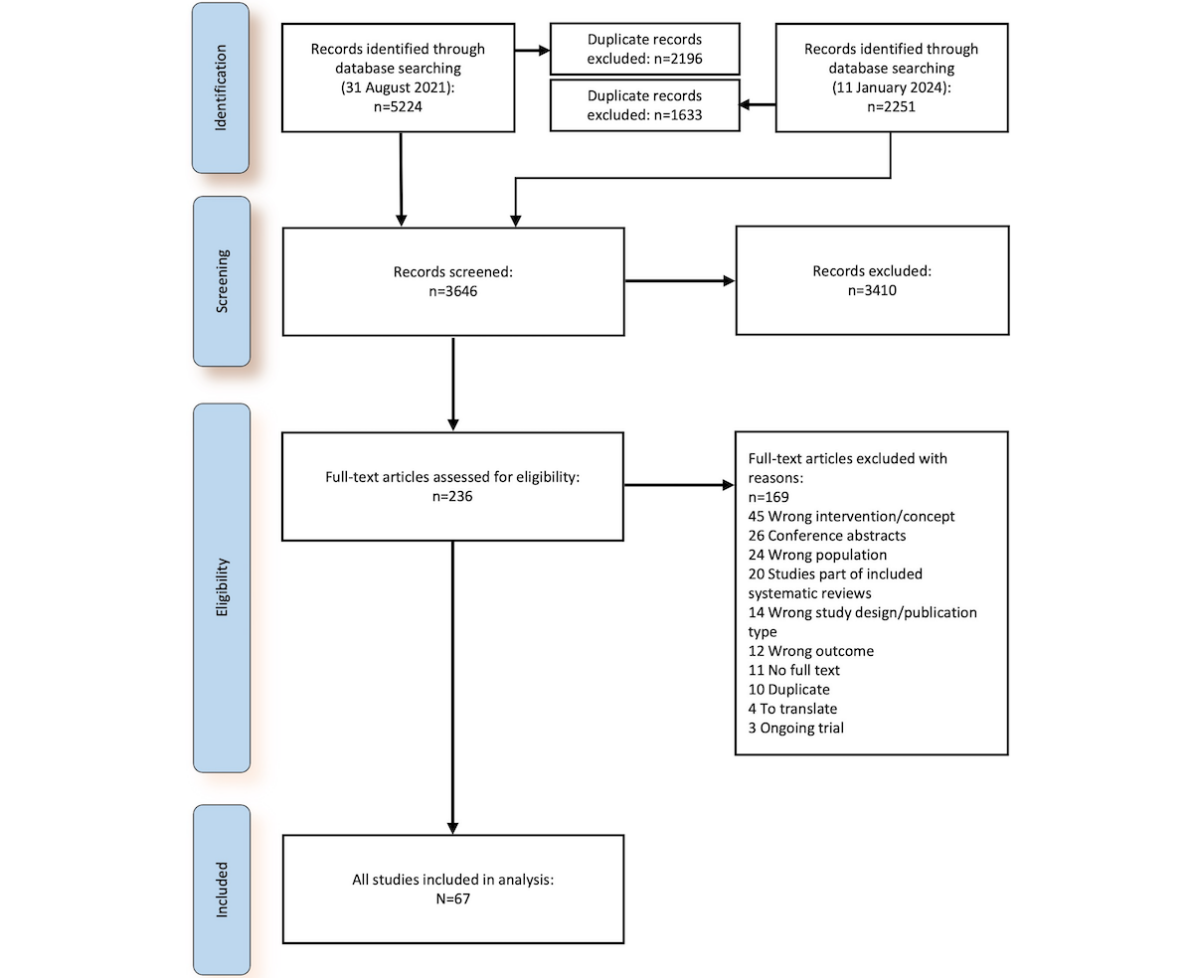
The PRISMA (Preferred Reporting Items for Systematic Reviews and Meta-Analyses) flow diagram.

We screened 3646 titles and abstracts and 236 full-text articles. We identified 6 systematic reviews and 61 primary studies (Figure 2) for inclusion. We divided primary studies into 6 different categories (themes) and described each category in more detail below. Table 2 gives an overview of the categories and references to further detail.

**Table 2:** Overview of categories into which included studies were divided.

Type of study		Articles		Further detail
Systematic reviews		n=6		Table 3
**Primary studies (N=61)**				
	Close- or household-contact investigations	n=34 (56%)	Multimedia Appendix 3 and Table 4	
	Outbreak investigations	n=17 (28%)	Table 5	
	Airline contact investigations	n=3 (5%)		
	Epidemiological analyses	n=5 (8%)		
	Public-private partnership program	n=1 (2%)		
	E-registry program	n=1 (2%)		

### Systematic Reviews

We identified 6 systematic reviews. Outcomes were descriptive and none of the reviews identified any randomized controlled trials. In 5 of the 6 reviews, the date of the last search was more than 5 years ago (Table 3). In reviews with the yield of TB disease as an outcome, the denominator was reported as the number of contacts evaluated or screened; however, specific definitions for “evaluated” or “screened” were not reported and the yield for a specific screening or diagnostic strategy is unknown.

**Table 3:** Overview of the included systematic reviews.

Review	Primary outcome	Date of last search
Abubakar [17]	Number of contacts screened, and number of individuals with TB^a^ infection and TB disease identified	November 2009
Fox et al [18]	Yield of TB disease and TB infection for both DS-TB^b^ and DR-TB^c^ source cases	October 2011
Shah et al [19]	Yield of TB infection and TB disease in contacts of DR-TB source cases	December 2011
Kodama et al [20]	Relative risk ratio of TB disease in DS-TB contacts compared with DR-TB contacts	Not reported
Svadzian et al [21]	Proportion of cases from those evaluated through universal testing (all individuals in the study received DST^d^) and those evaluated through selective testing (only the high-risk group received DST)	June 2019
Chiang et al [22]	Percentage of secondary cases whose *Mycobacterium tuberculosis* strains were resistant to the same drugs as strains from the index cases	July 2018

^a^TB: tuberculosis.

^b^DS-TB: drug-susceptible tuberculosis.

^c^DR-TB: drug-resistant tuberculosis.

^d^DST: drug-susceptibility testing.

### Primary Studies

We identified 61 primary studies that were not included in any of the above reviews. Primary studies were descriptive and included cross-sectional studies, prospective studies, and retrospective reviews of program data. No trials were identified. Thirty-four studies were close-contact or household-contact investigations, 17 were outbreak investigations, 3 were airline contact investigations, 5 were epidemiological analyses, 1 described a private-public partnership program, and 1 assessed the feasibility and acceptability of an e-registry program (Table 2). Case-finding pathways were seldom described clearly, for example, whether contacts were invited for screening regardless of symptoms (Figure 1, blue pathway), whether all contacts were screened for TB at home (Figure 1, orange pathway), or whether those who experienced TB symptoms were invited for further tests (Figure 1, black dashed pathway).

#### Close-Contact or Household-Contact Investigations

Countries where contact investigations were conducted included South Africa (n=6), India (n=5), Pakistan (n=4), Australia (n=2), the United States (n=1), Ethiopia (n=2), Myanmar (n=2), Thailand (n=1), France (n=1), Vietnam (n=1), Papua New Guinea (n=1), Armenia (n=1), the United Kingdom (n=1), Spain (n=1), South Korea (n=1), Oman (n=1), and Tajikistan (n=1). Two multicountry studies were conducted in Botswana, Brazil, Haiti, Kenya, Peru, South Africa, and Thailand. Data extraction from contact investigations was difficult due to incomplete reporting of relevant information, such as the total number of source cases or the number of cases tested for drug susceptibility (Table 4). Screening and diagnostic tools were not well reported and often lacked consistent or standardized use. Although investigations focused on contacts who had been exposed to DR-TB, drug-susceptibility testing (DST) was seldom reported. Case-finding pathways were also not clearly described. Some contacts were followed up over 1-2 years and some were only evaluated at baseline. Lack of or inconsistent reporting of this relevant data results in an unknown or inconsistent denominator when calculating the yield of screening the contacts of individuals with TB and makes it challenging to pool results or compare different case-finding strategies. There was also little consistency in the use of definitions. Source cases were often defined as “registered MDR-TB or extensively drug-resistant tuberculosis (XDR-TB) cases” without knowledge of how they were diagnosed. Several different definitions for “close contact” or “household contact” were reported. Some definitions were broad, for example, “people living with or having regular daily interaction with the MDR-TB source case” [23], while other definitions were more specific, for example, “a person who had shared the same enclosed living space for one or more nights a week, or for frequent or extended periods of time during the day, with the index patient during the 3 months before the current treatment episode began” [24-26]. The latter definition was used more often. See Multimedia Appendix 3 for more details.

**Table 4:** Data from DR-TB^a^ contact investigation studies.

Study	Source/Index cases			Contacts		Screening			TB^b^ diagnosis			DS-TB^c^ diagnosis		DR-TB diagnosis	
	Total identified	Studied	Total identified		Screened		Positive screen	Evaluated		Total TB	Diagnosed		Evaluated		Diagnosed
Mohammadi et al [27]	Not reported	13	Not reported		140		Not reported	Not reported		0	0		Not reported		0
Tuberculosis Research Centre, Indian Council of Medical Research [28]	Not reported	209 INH^d^ resistant at intake	779 at intake and 8358 over 15 years		Not reported		Not reported	Not reported		22 over 15 years of f/u^e^ and 260 per 100,000 person-years in INH-resistant HH^f^ contacts	18		22		4 INH resistant
Denholm et al [29]	47	47	570		49 LTBI^g^		Not reported	Not reported		2	Not reported		Not reported		2
Seddon et al [23]	Not reported	Not reported	281		228		Not reported (102 LTBI)	Not reported		15	Not reported		Not reported		Not reported
Adler-Shohet et al [30]	1	1	Not reported		118		31 TST^h^ pos^i^ (21 initially and 10 at repeat)	31 on LTBI treatment had 2-year f/u		0	0		0		0
Garcia-Prats et al [31]	1	1	38		34		None	Not reported		0	0		0		0
Titiyos et al [32]	508	508	155 family members in households of 29 symptomatic contacts		155		Unclear (29 symptomatic contacts were initially identified and evaluated)	Not reported		16	Not reported		Not reported		16 confirmed MDR-TB^j^
Arnold et al [33]	1	1	35		33		Not reported	Not reported		2 at baseline (2 in 2-year f/u)	0		Not reported		2 XDR^k^ (1 confirmed and 1 probable)
García et al [34]	1	1	39		39		19 Mantoux pos of which 1 had CXR^l^ changes	Not reported		5 (within 2-year f/u)	Not reported		4		4 INH resistant (same strain as index case)
Javaid et al [35]	200	154	Not reported		610		218 symptoms, 51 AFB^m^-positive, and Nr^n^ with abnormal CXR not reported	Not reported		51	10		Not reported		41
Fournier et al [36]	68	32	84		Not reported		Not reported	Not reported		Not reported	2		Not reported		3 MDR-TB
Golla et al [37]	Not reported	Not reported	Not reported		229		Not reported	226		15 (7 bacteriological)	Not reported		Not reported		Not reported
Lee et al [38]	1	1	7		6		2 asymptomatic with minimal nodules in baseline chest CT^o^ scan	2 followed up with chest CT scan		1	0		1		1
Chatla et al [39]	1602	1602	4858		4771		793	781		34	19		34		15
Dayal et al [40]	Not reported	43	Not reported		100		Not reported	100		41	Not reported		Not reported		Not reported
Hiruy et al [24]	111	111	340		331		20	20		9	1		20		8
Huerga et al [41]	265	111	198		150 at baseline and 138 at f/u		Not reported	Not reported		3 at baseline (none with f/u)	Not reported		Not reported		Not reported
Boonthanapat et al [42]	91	43	174		70 had screening records		3 abnormal CXRs	Not reported		1 identified, but no screening records for this one	Not reported		Not reported		Not reported
Hoang et al [43]	112	99	496		325 at baseline and 160 at f/u		36 symptoms, 12 abnormal CXR, and 27 at f/u	48 at baseline and 27 at f/u		1 (no TB at f/u)	1		48		0
Honjepari et al [44]	67	67 total (only 25 DR-TB)	697		635 total and 23 DR-TB contacts		156	114		9	5		Not reported		4 (2 bacteriologically and 2 clinically)
Kigozi et al [45]	Not reported	92	297 (only 6 contacts of MDR-TB cases)		259 (6)		102 (1)	48 (1)		17 (0)	Not reported		Not reported		Not reported
Phyo et al [26]	556	556	1908		1134		344 presumptive TB	186 presumptive TB and 213 others (399 in total)		27 (6 bacteriologically and 21 clinically)	15		20		5
Gupta et al [46]	308	284	1016		1007		228 signs and symptoms and 169/969 abnormal CXR	1007		121 (17 bacteriological)	11		16		5
Kyaw et al [25]	Not reported	210	Not reported		620		240 symptoms and Nr with abnormal CXR not reported	169 AFB/Xpert (71 symptomatic contacts did not receive AFB/Xpert)		24 (7 bacteriologically and 17 clinically)	22		43		2
Malik et al [47]	Not reported	100	800 (8 on TB treatment)		737		402 symptoms or <18 years or DM^p^ or HIV or low BMI^q^	326		3	Not reported		Not reported		Not reported
Paryani et al [48]	129	109	518 (22 with TB already on treatment and 2 diagnosed at baseline)		495 (400 entered f/u)		Not reported	Not reported		22 already on treatment (excluded), 2 diagnosed at baseline (excluded), and 14 at f/u	6		Not reported		6 pre-XDR, 1 XDR, and 1 missing DST^r^
Shadrach et al [49]	Not reported	87	Not reported		Not reported		285	271		97	35		Not reported		62
van de Water et al [50]	284	284	959		336		Not reported	Not reported		30	Not reported		Not reported		Not reported
Chang et al [51]	Not reported	55	247		215		8 abnormal CXR	Not reported		1	1		Not reported		Not reported
Kim et al [52]	305	152 RR-TB	1324		303 children <15 years		69 symptoms	93 smear microscopy, 55 CXR, and 93 culture or molecular test		4 bacteriologically and 49 clinically	Not reported		Not reported		Not reported
Ahmed et al [53]	329	324	1911		1734 symptom screen, 281 Xpert, and 123 CXR only		Not reported	All contacts were eligible for Xpert regardless of symptoms		20	0		20		2 clinically, 7 MDR/RR^s^ TB, and 11 pre-XDR TB
Ahmed and Dadlani [54]	470	100 MDR-TB and 370 DS-TB	830		830		218 symptoms	102 GeneXpert, 76 Smear microscopy, and 11 CXR		106	98		Not reported		8 MDR-TB
Apolisi et al [55]	Not reported	48	146		112		19 symptoms	55 CXR only, 19 CXR and sputum, and 1 sputum only		11 (2 bacteriologically and 9 clinically)	1 (bacteriologically)		Not reported		10 (1 indeterminate for rifampicin susceptibility and 9 clinically)
Rekart et al [56]	Not reported	830 RR-TB	Not reported		6654		269 symptoms	1549		49	1		28 had DST		47 RR (16 MDR, 12 XDR) and 1 INH resistant

^a^DR-TB: drug-resistant tuberculosis.

^b^TB: tuberculosis.

^c^DS-TB: drug-susceptible tuberculosis.

^d^INH: isoniazid.

^e^f/u: follow-up.

^f^HH: household.

^g^LTBI: latent TB infection.

^h^TST: tuberculin skin test.

^i^pos: positive.

^j^MDR-TB: multidrug-resistant tuberculosis.

^k^XDR: extensively drug-resistant.

^l^CXR: chest radiography.

^m^AFB: acid fast bacilli.

^n^nr: number.

^o^CT: computed tomography.

^p^DM: diabetes mellitus.

^q^BMI: body mass index.

^r^DST: drug susceptibility testing.

^s^RR: rifampicin resistant.

#### Outbreak Investigations

The Dictionary of Epidemiology defines an outbreak as “an epidemic limited to localized increase in the incidence of disease, e.g., village, town, or closed institution” [57]. In the included studies, it was not always reported whether the number of identified patients was more than expected over a particular period. It was not always clear if a study was indeed an investigation of an outbreak. Studies are summarized in Table 5. These were mostly in lower TB burden countries. They were all descriptive and focused on different aspects of an outbreak, for example, contact investigation and follow-up, preventive measures, and transmission chains.

**Table 5:** Overview of outbreak investigations.

Study	Country	Disease	Population	Cases that triggered a response	Focus of the paper
Valway et al [58]	United States (New York)	MDR-TB^a^	Inmates from a prison in Upstate New York	4 inmates from one prison were diagnosed in the summer of 1991	Transmission patterns and contact investigation results
Ridzon et al [59]	United States (California)	DR-TB^b^	California high-school students	4 students were diagnosed in spring 1993	Findings from an outbreak investigation
Breathnach et al [60]	United Kingdom (London)	MDR-TB	Patients who were HIV positive at St Thomas’ Hospital in London	8 patients were identified between 1995 and 1997	Epidemiology and control of the hospital outbreak
Holdsworth et al [61]	United Kingdom (London)	MDR-TB	Nosocomial outbreak at Guy’s and St Thomas’ NHS^c^ Trust	8 patients were identified in the summer of 1996	Management of public relations following the outbreak
Moro et al [62]	Italy	MDR-TB	Patients infected by HIV and hospitalized in an HIV ward in Milan, Italy	33 patients were diagnosed between October 1992 and February 1994	Risk factors for transmission and the effectiveness of infection control measures
Schmid et al [63]	Austria	MDR-TB	Refugees in Austria	In 2005-2006 the Austrian laboratory for TB^d^ identified 4 MDR-TB cases with similar genotypes	The chain of transmission
Asghar et al [64]	United States (Florida)	*Mycobacterium tuberculosis* resistant to isoniazid	HIV-positive, rock cocaine (crack) users who lived in low-income neighborhoods in Miami	18 cases with matching spoligotypes were identified between January 2004 and May 2005	Transmission patterns and recommendations for TB control in this population
Fred et al [65]	Federated States of Micronesia	MDR-TB	Cluster of patients with MDR-TB in Chuuk State	A cluster of 5 patients were identified in May 2008	Contact tracing and control measures
Chee et al [66]	Singapore	MDR-TB	LAN^e^ gaming centers	In 2012, 5 men who attended 2 LAN gaming centers were diagnosed with MDR-TB	Highlights gaming centers as potential hotspots for TB transmission and notes challenges when conducting contact-tracing investigations
Norheim et al [67]	Norway	Streptomycin resistant TB	Students attending training sessions at an educational institution in Oslo, Norway	3 students were identified within one week in April 2013	Transmission patterns linking data from contact tracing to data from WGS^f^
Ho et al [68]	Singapore	MDR-TB	Residents of an 11-storey apartment block	6 residents were identified between February 2012 and May 2016	The cluster investigation and results from mass screening
Popovici et al [69]	Romania	XDR-TB^g^	Foreign medical students at a Romanian university	A cluster of 3 patients was identified in October 2015	Results from contact investigation and the efforts to identify the source case
Zhang et al [70]	China	MDR-TB	School in Zhejiang Province	A student was diagnosed in May 2014	Results from classmate contact investigation
Li et al [71]	China	MDR-TB	Senior high school	A female student diagnosed in March 2020	Results from household, classmate, and faculty investigations
Kobayashi et al [72]	Japan	MDR-TB	Japanese language school in Tokyo	A student was diagnosed in September 2019	Results from analysis of outbreak cases
Wu et al [73]	China	RR-TB^h^	Middle school in Jiangsu Province	Unclear. 12 patients were diagnosed with TB of whom 6 were RR-TB.	Describe characteristics and epidemiology of outbreak and suggestions for prevention and control of school TB
Groenweghe et al [74]	United States (Kansas)	MDR-TB	Households from the same apartment complex and their contacts	The first person identified was an infant hospitalized in November 2021. An investigation identified 4 additional household members with MDR-TB.	Public Health Response and results from contact investigations

^a^MDR-TB: multidrug-resistant tuberculosis.

^b^DR-TB: drug-resistant tuberculosis.

^c^NHS: National Health Services.

^d^TB: tuberculosis.

^e^LAN: local area network.

^f^WGS: whole-genome sequencing.

^g^XDR-TB: extensively drug-resistant tuberculosis.

^h^RR-TB: rifampicin-resistant tuberculosis.

#### Airline Contact Investigations

Three studies described airline DR-TB contact investigation. An der Heiden et al [75] investigated passengers and crew members after exposure to an individual with XDR-TB. The response rate was 83%. No secondary TB cases were reported and 1 individual with TB infection, probably newly acquired, was identified. Kornylo-Duong et al [76] evaluated passenger contacts of individuals with MDR-TB. More than 65% were lost to follow-up. No secondary TB cases were reported. Eight contacts tested positive for latent tuberculosis infection (LTBI); however, it was unknown if these contacts were recent converters as they might have acquired LTBI from their countries of residence. Glasauer et al [77] analyzed international contact tracing notifications received by Germany from other countries, with a focus on air travel. The high variability in the completeness of contact tracing information made analyses a challenge.

#### Epidemiological Analyses

Nitta et al [78], Anderson et al [79], de Vries et al [80], and Suppli et al [81] described transmission patterns of DR-TB in Los Angeles County (1993-1998), the United Kingdom (2004-2007), and the Netherlands (2010-2019 and 2018-2019), respectively. Intervention strategies to find those with TB disease are mentioned, but not described in detail. Villa et al [82] described a cluster of 16 pre-XDR and XDR-TB cases in Italy between 2016 and 2020 as well as the role of whole-genome sequencing in TB surveillance.

#### Public-Private Partnership Program

Joloba et al [83] described a program to improve MDR-TB detection by improving access to rapid and reliable DST, redesigning the TB specimen transport network, and training health care workers in Uganda. This study enhanced the care-seeking pathway (Figure 1, green dashed pathway) to specifically improve MDR-TB diagnosis and no screening took place.

#### E-registry Program

Naker et al [84] described a qualitative study in Mongolia to assess the feasibility and acceptability of an e-registry tool to simplify the systematic screening of MDR-TB contacts. Of 42 index cases invited to take part in the pilot study, 10 declined participation due to concerns about data security.

## Discussion

### Key Findings

This scoping review charts the existing literature on DR-TB case finding. More than 60% of identified studies described DR-TB contact investigations. Included studies were all descriptive and no trials were identified. There is a lack of primary studies for inclusion in systematic reviews assessing the effectiveness, cost-effectiveness, acceptability, and feasibility of different DR-TB case-finding strategies. Case-finding strategies were not always reported in enough detail to deduce the specific pathways in our systems-based logic model (Figure 1), for example, whether symptomatic contacts were invited to a TB diagnostic service or whether contacts were screened at home or invited for screening at a health facility; however, except for target group differences, for example, contacts of DR-TB source cases compared with DS-TB source cases, and DST when presumptive DR-TB is identified, we did not note any differences between DR-TB case-finding and DS-TB case-finding strategies. Information on factors that may influence the yield of TB disease, like the number of contacts identified, screened, and evaluated, when these contacts were evaluated (baseline or follow-up), and which screening and diagnostic tools used [85] were seldom reported in detail.

### Previous Work

Although conclusions about the most effective DR-TB case-finding strategy cannot be drawn, several reviews looked at the possible effects of active TB case finding (screening pathways in Figure 1) compared with passive TB case finding (care-seeking pathways in Figure 1) in general. Two Cochrane reviews failed to identify any studies for inclusion. Fox et al [86] aimed to compare the diagnostic yield of TB disease between active case finding and passive case finding in TB contacts but did not identify any trials for inclusion in the review. Braganza Menezes et al [87] also could not identify trials for inclusion in a review aiming to assess the effectiveness of novel methods, for example, social network analysis, of contact tracing versus the current standard of care to identify individuals with TB infection or TB disease. A review by Kranzer et al [88] included observational studies and concluded that screening compared with standard care increases the number of patients with TB disease found in the short term, but that it is unknown whether it impacts TB epidemiology. This finding was underpinned by another Cochrane review. Mhimbira et al [5] found that active case-finding strategies may result in increased case finding in the short term, but long-term outcomes were lacking. Except for the unknown effect of TB screening on TB epidemiology, the effect of screening on individual outcomes had been studied by Telisinghe et al [89] and Kranzer et al [88]. These reviews found limited patient outcome data and no difference in treatment outcomes between active and passive case findings. While it is known that screening may increase the number of identified cases, it is unclear if TB screening makes a difference to TB epidemiology and individual outcomes compared with passive case finding.

### Implications for Research

There is a need for standardization of terminology, design, and reporting of DR-TB case-finding studies, especially contact investigation studies. Future research should focus on clear definitions, methodology, and detailed descriptions of all intervention components. There is also a need for well-conducted randomized controlled trials assessing the effect of active case finding on individual outcomes and long-term TB epidemiological outcomes.

### Strengths and Limitations

The strengths of our review included a thorough search strategy and the use of a systems-based logic model. Nevertheless, there were some limitations. We searched several databases with no date or language restriction, but we did not translate all full-text articles. Studies that were not translated (n=4) are reported in the list of excluded studies (Multimedia Appendix 4) and from the translated abstracts it seems that similar study designs were found to those of included contact investigation studies. Our review excluded patients with TB symptoms seeking care, patients diagnosed with TB, and laboratory samples or laboratory isolates because the focus of this review was on active case finding. It should therefore be noted that studies that investigated strategies to improve identification of DR-TB after clinical identification of presumptive DR-TB cases, or studies that screened laboratory samples were not part of this review. Furthermore, for collating, summarizing, and reporting the results, we initially envisaged using our systems-based logic model as a framework to describe different case-finding strategies and resulting pathways. However, reporting was incomplete and inconsistent, and we were not able to describe pathways in detail. Nevertheless, the logic model guided our interpretation of whether a case-finding study involved screening or not. Finally, for contact investigation studies, we included studies that reported on the number of individuals with TB disease diagnosed, even if the study focused on LTBI testing and treatment. This might be a reason why the screening and diagnostic pathways were not always reported in detail. However, it is important to note that in contact investigation studies, the active case-finding component and the LTBI treatment component are both important aspects of early case finding and prevention.

### Conclusions

Existing descriptive reviews can be updated, but there is a dearth of knowledge on the effectiveness, cost-effectiveness, acceptability, and feasibility of DR-TB case-finding strategies to inform policy and practice. There is also a need for standardization of terminology, design, and reporting of DR-TB case-finding studies, especially contact investigation studies, to decrease a large amount of research waste and increase the number of studies that could be synthesized and meta-analyzed in high-impact systematic reviews in the future [90].

## Appendix

Multimedia Appendix 1 PRISMA-ScR (Preferred Reporting Items for Systematic Reviews and Meta-Analyses extension for Scoping Reviews) checklist.

Multimedia Appendix 2 Detailed search strategy.

Multimedia Appendix 3 Characteristics of drug-resistant TB contact investigation studies.

Multimedia Appendix 4 List of excluded studies with reasons.

## Abbreviations

DR-TB: drug-resistant tuberculosis
DS-TB: drug-susceptible tuberculosis
DST: drug-susceptibility testing
LTBI: latent tuberculosis infection
MDR-TB: multidrug-resistant tuberculosis
PRISMA-ScR: Preferred Reporting Items for Systematic Reviews and Meta-Analyses extension for Scoping Reviews
TB: tuberculosis
XDR-TB: extensively drug-resistant tuberculosis

## Data Availability

All data analyzed during this study are included in this published article and its Multimedia Appendices.
