# Supplementary material for: Drug-Resistant Tuberculosis Case-Finding Strategies: Scoping Review
Source: JMIR Public Health Surveill. 2024 Jun 26;10:e46137. doi: 10.2196/46137 (PMC11237795; doi:10.2196/46137)
Supplement: Multimedia Appendix 2 [file publichealth_v10i1e46137_app2.doc]

**31 AUGUST 2021**

Medline PubMed

#1 "Tuberculosis, Multidrug-Resistant"[Mesh]

#2 "Extensively Drug-Resistant Tuberculosis"[Mesh]

#3 ("tuberculosis"[MeSH] OR "Mycobacterium tuberculosis"[MeSH] OR TB[Title/Abstract] OR tuberculosis[Title/Abstract]) AND (drug resist*[Title/Abstract] OR MDR[Title/Abstract] OR DR[Title/Abstract] OR XDR[Title/Abstract])

#4 DR-TB[Title/Abstract] OR MDR-TB[Title/Abstract] OR XDR-TB[Title/Abstract]

#5 Case* detection[Title/Abstract]

#6 Case* finding[Title/Abstract]

#7 Screening[Title/Abstract]

#8 contact investigation[Title/Abstract] OR contact tracing[Title/Abstract] OR contact finding[Title/Abstract]

#9 #1 OR #2 OR #3 OR #4

#10 #5 OR #6 OR #7 OR #8

#11 #9 AND #10

Embase

Embase 1947-Present, updated daily

1 multidrug resistant tuberculosis/

2 extensively drug resistant tuberculosis/

3 Mycobacterium tuberculosis/ or tuberculosis/

4 (TB or tuberculosis).tw.

5 drug resistance.mp. or drug resistance/

6 drug resistant.tw.

7 3 or 4

8 5 or 6

9 7 and 8

10 (DR-TB or MDR-TB or XDR-TB).tw.

11 1 or 2 or 9 or 10

12 case detection.mp.

13 case finding.mp. or case finding/

14 screening.tw.

15 contact investigation.mp.

16 contact tracing.mp. or contact examination/

17 contact finding.mp.

18 12 or 13 or 14 or 15 or 16 or 17

19 11 and 18

20 (rat or rats or mouse or mice or swine or porcine or murine or sheep or lambs or pigs or piglets or rabbit or rabbits or cat or cats or dog or dogs or cattle or bovine or monkey or monkeys or trout or marmoset*).ti. and animal experiment/

21 Animal experiment/ not (human experiment/ or human/)

22 20 or 21

23 19 not 22

The Cochrane Library

ID Search Hits

#1 MeSH descriptor: [Tuberculosis, Multidrug-Resistant] explode all trees

#2 MeSH descriptor: [Extensively Drug-Resistant Tuberculosis] explode all trees

#3 MeSH descriptor: [Tuberculosis] explode all trees

#4 MeSH descriptor: [Mycobacterium tuberculosis] explode all trees

#5 (TB):ti,ab,kw

#6 (tuberculosis):ti,ab,kw

#7 #3 OR #4 OR #5 OR #6

#8 (drug resist*):ti,ab,kw

#9 (MDR):ti,ab,kw

#10 (DR):ti,ab,kw

#11 (XDR):ti,ab,kw

#12 #8 OR #9 OR #10 OR #11

#13 #7 AND #12

#14 (DR-TB):ti,ab,kw

#15 (MDR-TB):ti,ab,kw

#16 (XDR-TB):ti,ab,kw

#17 #1 OR #2 OR #13 OR #14 OR #15 OR #16

#18 (Case* detection):ti,ab,kw

#19 (Case* finding):ti,ab,kw

#20 (Screening):ti,ab,kw

#21 (contact investigation):ti,ab,kw

#22 (contact tracing):ti,ab,kw

#23 (contact finding):ti,ab,kw

#24 #18 OR #19 OR #20 #21 OR #22 OR #23

#25 #17 AND #24 in Cochrane Reviews, Cochrane Protocols, Trials

Africa-Wide Information (EBSCOhost)

| **#** | **Query** |
| --- | --- |
| S11 | S5 AND S10 |
| S10 | S6 OR S7 OR S8 OR S9 |
| S9 | TI ( contact investigation OR contact tracing OR contact finding ) OR AB ( contact investigation OR contact tracing OR contact finding ) |
| S8 | TI screening OR AB screening |
| S7 | TI ( case finding or cases finding ) OR AB ( case finding or cases finding ) |
| S6 | TI ( case detection or cases detection ) OR AB ( case detection or cases detection ) |
| S5 | S3 OR S4 |
| S4 | TI ( DR-TB OR MDR-TB OR XDR-TB ) OR AB ( DR-TB OR MDR-TB OR XDR-TB ) |
| S3 | S1 AND S2 |
| S2 | TI ( drug resistant OR drug resistance ) OR AB ( drug resistant OR drug resistance ) |
| S1 | TI ( tuberculosis OR TB ) OR AB ( tuberculosis OR TB ) |

CINAHL (EBSCOhost)

| **#** | **Query** |
| --- | --- |
| S22 | S12 AND S21 |
| S21 | S13 OR S14 OR S15 OR S16 OR S17 OR S18 OR S19 OR S20 |
| S20 | TI contact investigation OR contact tracing OR contact finding |
| S19 | AB contact investigation OR contact tracing OR contact finding |
| S18 | AB screening |
| S17 | TI screening |
| S16 | TI case finding or cases finding |
| S15 | AB case finding or cases finding |
| S14 | AB case detection or cases detection |
| S13 | TI case detection or cases detection |
| S12 | S1 OR S2 OR S8 OR S9 OR S11 |
| S11 | S5 AND S10 |
| S10 | S6 OR S7 |
| S9 | TI DR-TB OR MDR-TB OR XDR-TB |
| S8 | AB DR-TB OR MDR-TB OR XDR-TB |
| S7 | AB drug resistant OR drug resistance |
| S6 | TI drug resistant OR drug resistance |
| S5 | S3 OR S4 |
| S4 | AB tuberculosis OR tb |
| S3 | TI tuberculosis OR tb |
| S2 | MJ tuberculosis drug resistant |
| S1 | MJ tuberculosis multidrug resistant |

Epistemonikos

(title:((title:((tuberculosis OR TB) AND (drug resistant OR drug resistance) AND (case detection OR case finding OR screening OR contact investigation OR contact tracing OR contact finding)) OR abstract:((tuberculosis OR TB) AND (drug resistant OR drug resistance) AND (case detection OR case finding OR screening OR contact investigation OR contact tracing OR contact finding)))) OR abstract:((title:((tuberculosis OR TB) AND (drug resistant OR drug resistance) AND (case detection OR case finding OR screening OR contact investigation OR contact tracing OR contact finding)) OR abstract:((tuberculosis OR TB) AND (drug resistant OR drug resistance) AND (case detection OR case finding OR screening OR contact investigation OR contact tracing OR contact finding)))))

PROSPERO

**(tuberculosis OR TB) AND (drug resistant OR drug resistance) AND (case detection OR case finding OR screening OR contact investigation OR contact tracing OR contact finding)**

**11 JANUARY 2024**

| **Pubmed (Medline)** | |
| --- | --- |
| Search number | Query |
| 1 | "Tuberculosis, Multidrug-Resistant"[Mesh] |
| 2 | "Extensively Drug-Resistant Tuberculosis"[Mesh] |
| 3 | tuberculosis[MeSH Terms] |
| 4 | Mycobacterium tuberculosis[MeSH Terms] |
| 5 | TB[Text Word] OR tuberculosis[Text Word] |
| 6 | drug resist*[Text Word] OR multidrug* resist*[Text Word] OR treatment resist*[Text Word] OR refractory[Text Word] |
| 7 | ((TB[Text Word] OR tuberculosis[Text Word]) OR (Mycobacterium tuberculosis[MeSH Terms])) OR (tuberculosis[MeSH Terms]) |
| 8 | (((TB[Text Word] OR tuberculosis[Text Word]) OR (Mycobacterium tuberculosis[MeSH Terms])) OR (tuberculosis[MeSH Terms])) AND (drug resist*[Text Word] OR multidrug* resist*[Text Word] OR treatment resist*[Text Word] OR refractory[Text Word]) |
| 9 | DR-TB[Text Word] OR MDR-TB[Text Word] OR XDR-TB[Text Word] |
| 10 | ((((DR-TB[Text Word] OR MDR-TB[Text Word] OR XDR-TB[Text Word])) OR ((((TB[Text Word] OR tuberculosis[Text Word]) OR (Mycobacterium tuberculosis[MeSH Terms])) OR (tuberculosis[MeSH Terms])) AND (drug resist*[Text Word] OR multidrug* resist*[Text Word] OR treatment resist*[Text Word] OR refractory[Text Word]))) OR ("Extensively Drug-Resistant Tuberculosis"[Mesh])) OR ("Tuberculosis, Multidrug-Resistant"[Mesh]) |
| 12 | Case* and detect*[Text Word] |
| 15 | case finding[Text Word] |
| 16 | case* finding[Title/Abstract] |
| 17 | case detect*[Title/Abstract] |
| 18 | "Contact Tracing"[Mesh] |
| 19 | screening[Title/Abstract] |
| 20 | contact* investigation[Title/Abstract] |
| 21 | contact* tracing[Title/Abstract] |
| 22 | contact* finding[Title/Abstract] |
| 25 | contact* screening[Text Word] |
| 26 | ((((((((contact* screening[Text Word]) OR (contact* finding[Title/Abstract])) OR (contact* tracing[Title/Abstract])) OR (contact* investigation[Title/Abstract])) OR ("Contact Tracing"[Mesh])) OR (case detect*[Title/Abstract])) OR (case* finding[Title/Abstract])) OR (case finding[Text Word])) OR (Case* and detect*[Text Word]) |
| 27 | (((((((((contact* screening[Text Word]) OR (contact* finding[Title/Abstract])) OR (contact* tracing[Title/Abstract])) OR (contact* investigation[Title/Abstract])) OR ("Contact Tracing"[Mesh])) OR (case detect*[Title/Abstract])) OR (case* finding[Title/Abstract])) OR (case finding[Text Word])) OR (Case* and detect*[Text Word])) AND (((((DR-TB[Text Word] OR MDR-TB[Text Word] OR XDR-TB[Text Word])) OR ((((TB[Text Word] OR tuberculosis[Text Word]) OR (Mycobacterium tuberculosis[MeSH Terms])) OR (tuberculosis[MeSH Terms])) AND (drug resist*[Text Word] OR multidrug* resist*[Text Word] OR treatment resist*[Text Word] OR refractory[Text Word]))) OR ("Extensively Drug-Resistant Tuberculosis"[Mesh])) OR ("Tuberculosis, Multidrug-Resistant"[Mesh])) |

**Embase** 1947-Present, updated daily

1 multidrug resistant tuberculosis/ or drug resistant tuberculosis/

2 extensively drug resistant tuberculosis/

3 tuberculosis/ or Mycobacterium tuberculosis/

4 (tuberculosis or TB).ti,ab.

5 (drug* adj2 resist*).mp.

6 (multidrug* adj2 resist*).mp.

7 refractory.tw.

8 (treatment adj2 resist*).mp.

9 3 or 4

10 drug resistance/

11 (DR-TB or MDR-TB or XDR-TB).mp.

12 5 or 6 or 7 or 8 or 10

13 9 and 12

14 1 or 2 or 11 or 13

15 ((case or cases) adj2 detect*).mp.

16 case finding/

17 (case* adj2 finding).mp

18 (case* adj2 screening).mp.

19 contact examination/ or contact* screening.mp.

20 (contact* adj2 investigation).mp.

21 contact* tracing.mp.

22 (contact* adj2 tracing).mp.

23 15 or 16 or 17 or 18 or 19 or 20 or 21 or 22

24 14 and 23

**Cochrane Database of Systematic Reviews**

Issue 1 of 12, January 2024

#1 MeSH descriptor: [Tuberculosis, Multidrug-Resistant] explode all trees

#2 MeSH descriptor: [Extensively Drug-Resistant Tuberculosis] explode all trees

#3 ((Tuberculosis or Mycobacterium tuberculosis) near/3 (drug resistant or multidrug resistant)):ti,ab,kw

#4 ((Tuberculosis or Mycobacterium tuberculosis) near/3 (drug resistant or multidrug resistance)):ti,ab,kw

#5 refractory near/2 (tuberculosis or TB)

#6 ((MDR or DR or XDR) near/2 (tuberculosis or TB)):ti,ab,kw

#7 #1 or #21 or #3 or #4 or #5 or #6

#8 MeSH descriptor: [Contact Tracing] explode all trees

#9 case* NEXT finding*

#10 cases* NEXT detection

#11 contact* NEXT screening

#12 contact NEXT tracing

#13 contact* NEXT investigation

#14 #8 or #9 or #10 or #11 or #12 or #13

#15 #7 and #14

Interface - EBSCOhost Research Databases

**Database - CINAHL**

| **#** | **Query** |
| --- | --- |
| S9 | S4 AND S8 |
| S8 | S5 OR S6 OR S7 |
| S7 | TX contact* screening OR TX contact* finding OR TX contact* investigat* |
| S6 | MH contact tracing |
| S5 | TX case* detection OR TX case* finding |
| S4 | S1 OR S2 OR S3 |
| S3 | TX DR-TB OR MDR-TB OR XDR-TB |
| S2 | TX ( tuberculosis or tb or mycobacterium tuberculosis ) AND TX ( drug* resist* OR multidrug* resist* OR treatment* resist* OR refractory ) |
| S1 | MH tuberculosis, multidrug-resistant OR MH extensively drug-resistant tuberculosis |

**Epistemonikos**

(title:(((tuberculosis OR TB) AND (drug resistant OR drug resistance OR multidrug resistant OR multidrug resistance) AND (case detection OR case finding OR screening OR contact investigation OR contact tracing OR contact finding))) OR abstract:(((tuberculosis OR TB) AND (drug resistant OR drug resistance OR multidrug resistant OR multidrug resistance) AND (case detection OR case finding OR screening OR contact investigation OR contact tracing OR contact finding))))

**PROSPERO**

(tuberculosis OR TB) AND (drug resistant OR drug resistance) AND (case detection OR case finding OR screening OR contact investigation OR contact tracing OR contact finding)
