# Supplementary material for: Drug-Resistant Tuberculosis Case-Finding Strategies: Scoping Review
Source: JMIR Public Health Surveill. 2024 Jun 26;10:e46137. doi: 10.2196/46137 (PMC11237795; doi:10.2196/46137)
Supplement: Multimedia Appendix 3 [file publichealth_v10i1e46137_app3.doc]

| **Study** | **Country** | **Study design** | **Time of study** | **Aim of study/ Outcomes assessed** | **Definitions used for “contact”** |
| --- | --- | --- | --- | --- | --- |
| Mohammadi et al [26] | Oman | Retrospective | January 2000 to October 2005 | In this study in Oman we sought to determine the epidemiological and clinical factors leading to acquisition of MDR-TB, the components of effective therapy and the economic repercussions of treatment | Close contacts were defined as household members, as well as work or school colleagues having prolonged, significant contact with an index case (i.e. eating and/or sleeping in the same room) |
| Tuberculosis Research Centre, Indian Council of Medical Research [27] | India | Prospective | Data from RCT done in 1968 | To compare the risk to household contacts of isoniazid (INH) susceptible and INH-resistant cases of tuberculosis (TB) in a rural community in South India. This was a re-analysis of an RCT to assess the efficacy of BCG vaccine which was done in 1968 with 15 years of follow-up. | A household was defined as a group of persons living together and sharing food from the same kitchen. |
| Denholm et al [28] | Australia | Retrospective | 1995 to 2010 | To perform long-term follow-up of MDRTB contacts and review individual outcomes and management approaches. | ‘Contacts’ are considered to be those with more than 8 h of cumulative exposure to potentially infective people (i.e., those with pulmonary disease, whether smear-positive or -negative) |
| Seddon et al [22] | South Africa | Cross-sectional | May 2010 through April 2011 | This study aimed to determine risk factors for M. tuberculosis infection and disease in children following exposure to adults with multidrug-resistant (MDR) tuberculosis (TB). | All children aged < 5 years, routinely referred per local guidelines to the provincial specialist MDR-TB clinic, Western Cape Province, South Africa, following identification as contacts of adult MDR-TB source cases, were eligible for enrolment from May 2010 through April 2011 |
| Adler-Shohet et al [29] | USA | Retrospective | Not reported | A teacher in California developed MDR-TB exposing dozens of children. This retrospective chart review describes our experience with the contact investigation and treatment of those child contacts. | A contact was defined as a child who spent ≥ 8 cumulative hours in either the same room as the case or the connected classroom. |
| Garcia-Prats et al [30] | South Africa | Retrospective | 2012 | To describe the results of a contact investigation of children exposed to an adult with DRTB at a South African home-based day care centre. | Age, 15 years, documented exposure to the index case at the day care centre |
| Titiyos et al [31] | Ethiopia | Retrospective | February 2013 to April  2013 | The yield of screening symptomatic contacts of multidrug‑resistant tuberculosis cases at a tertiary hospital in Addis Ababa | A person who shared the same enclosed living space |
| Arnold et al [32] | UK | Retrospective | Index case presented April 2013 | This paper outlines the outcomes of the contact tracing investigation of an infectious XDR-TB case in London and describes how early WGS assisted in rapid individualized drug treatment and identification of further linked cases*.* | Contacts were defined as household residents if they had lived with the index case whilst infectious. The house of the index case was the hub of a complex social network, and a ‘concentric circles approach’ was used to broaden the investigation.17 Persons with prolonged contact with the index case either through regular visits to the index case’s house (house social) or through work (work contacts) were also screened. When further cases were identified the process was repeated and their close contacts were screened. |
| Hernan-Garcia et al [33] | Spain | Cross-sectional | Index case presented Jan 2012 | Contact tracing of a case of pulmonary tuberculosis was performed in a Bolivian patient. | Contacts were classified as high priority (individuals with contact >6 h/day, children younger than 5 years, immunocompromised individuals), medium priority (individuals with daily contact, but <6 h/day), and low priority (sporadic contact) |
| Javaid et al [34] | Pakistan | Cross-sectional | May 2012 to May 2015 | To assess the profile of TB/multidrug-resistant TB (MDR-TB) among  household contacts of MDR-TB patients. | Household contacts were defined as individuals who had shared the same kitchen and sleeping area as the index case for at least 3 months before the diagnosis of the index case, and included spouses, children, parents, siblings and other relatives (uncles, grandfathers, cousins) |
| Fournier et al [35] | France | Prospective | Jan 2010 to Sep 2013 | We performed a study to investigate if this increase of MDR-TB index cases is accompanied by increased transmission. | Conducted contact tracing for all patients with MDR-TB and XDR-TB who were either living in Paris themselves or had contacts living in Paris.  Of the 84 contacts, 21 (25%) were living in the same house as the index case, 21 (25%) had contacts through collective accommodation, 17 (20%) were friends or family, and 25 (30%) were professional contacts. |
| Golla et al [36] | South Africa | Cross-sectional | August 2008 to June 2011 | We compared the risk of TB infection and TB disease in young child household contacts of adults with confirmed DS-TB and MDR-TB | Children were classified as being a household contact based on a definition of household which was developed and validated in the study setting, namely all dwellings on the same plot of land that share the same residential address |
| Lee et al [37] | Korea | Retrospective | Not reported | We preliminarily introduced submillisievert chest computed tomography (CT) scan (effective dose, 0.19–0.25 millisievert) in a contact investigation of multi-drug resistant (MDR)-TB. | Individuals older than 20 years old, with house­hold contact or working in the same room for more than 8 hours per day |
| Chatla et al [38] | India | Prospective | Dec 2013 to Jan 2014 | We undertook this study to measure the extent of TB symptoms developed among the household contact of the known MDR-TB patients treated under RNTCP and examine the symptomatic patients to measure the extent of Rifampicin sensitive TB (RS-TB) or Rifampicin resistance TB (RR-TB) among them, bacteriologically confirmed using Xpert MTB-Rif. | All household contacts of MDR-T patients on active care were identified and included in the study. |
| Dayal et al [39] | India | Cross-sectional | March 2015  through September 2016 | To find out the prevalence of latent tuberculosis (TB) infection and TB disease among pediatric household contacts of adult drug resistant (MDR) and drug susceptible (DS) TB patients and to identify the risk factors for occurrence of TB infection in the contacts*.* | Pediatric household contacts (less than 15 y age) of adult TB patients (both MDR and DS) were included in the study. Pediatric house hold contacts were defined as children who shared the same kitchen and sleeping area as the index case for at least 3 mo before the start of therapy of the index case. |
| Hiruy et al [23] | Ethiopia | Cross-sectional | 2015 | This study compared the yield of tuberculosis (TB) among contacts of multidrug-resistant tuberculosis (MDR-TB) index cases with that in drug-sensitive TB (DS-TB) index cases in a program setting. | A household contact was defined as a person who had shared the same enclosed living space for one or more nights a week, or for frequent or extended periods of time during the day, with the index patient during the 3 months before the current treatment episode began |
| Huerga et al [40] | Armenia | Prospective | June 2012 to December  2016 | We aimed to measure the prevalence and incidence of latent tuberculosis infection (LTBI) and tuberculosis (TB) disease in children in close contact with patients with drug-resistant TB (DR-TB) in a country with high DR-TB prevalence | A paediatric contact was defined as a child <15 years old living in the household of the index case or who had more than 7 days of contact for at least 4 hours per day during the 6 months prior to the index case registration. |
| Boonthanapat et al [41] | Thailand | Cross-sectional | October 2012 and September  2015 | We therefore conducted this study to explore the  feasibility and usefulness of Social Network Analysis (SNA) with the specific aims of  (i) describing the characteristics of the targeted MDR cases and their contacts and the services they received  and (ii) determining the prioritised contacts by applying  the SNA. | A ‘contact’ was defined as a person who was nominated by a case as  someone with whom the case had contacted during 2 years before starting MDR-TB treatment up to 2 months after. The “contacts” were divided into three exclusive groups: (i) household contact, (ii) close contact  and (iii) intermittent contact. The household contact was a person who shared enclosed living space with the index case. The close contact was an individual who shared activities such as working or studying with the index case for at least 8 h a day. The last group, the intermittent contact, was a person who contacted with the case for less than 8 h per day but more than 120 h per month in average. |
| Hoang et al [42] | Vietnam | Prospective | Index cases identified between Oct 2013 and April 2015 | We assessed the added value of active contact tracing within and  beyond households using social network questionnaires to identify close contacts of MDR-TB patients in Vietnam. | Their defined contacts (household contacts or contacts outside the household, either named by patients or from eligible places) during the 3 months preceding MDR-TB diagnosis were eligible for enrolment as contacts. Eligible high-risk places were physically enclosed spaces where the MDR-TB index case spent an average of at least 4 h a day for at least 14 days, or a cumulative total average duration of at least 8 h per week for at least 8 weeks in 3 months prior MDR-TB diagnosis. For children who were less than 18 years old, information was obtained from their parents or responsible family members. |
| Honjepari et al [43] | Papua New Guinea | Retrospective | 2016 to 2018 | We describe the implementation of the Daru household contact screening programme from 2016 to March 2018, and evaluate case finding and the provision of IPT to eligible contacts. | A household contact was defined as any person who shared the same enclosed living space with the index case for at least 1 month before diagnosis. |
| Kigozi et al [44] | South Africa |  | Sep through Oct 2016 | In order to inform and motivate scale-up, this pilot study investigated the effectiveness of systematic household contact investigation when targeting the World Health Organization’s (WHO) recommended categories of infectious index cases (including MDR TB cases. | Household contacts of any age were included in the study if they were not receiving TB treatment at the time of the home visit, had spent at least three months under the same roof as the infectious TB index case, had consented to the study, and were available for interviews during the household visits. |
| Phyo et al [25] | Myanmar | Prospective (mixed methods) | Jan 2018 to June 2019 | We undertook a mixed-methods operational research study with the following objectives: (1) Among the household contacts of MDR-TB patients registered from January 2018 to June 2019, to  assess (i) the number and proportion of presumptive TB patients identified, investigated, diagnosed, and treated for TB; (ii) demographic and clinical factors associated with getting or not getting investigated; and (iii) the median duration between the various steps in the cascade. (2) To explore the barriers in implementing contact investigation from the perspective of household contacts and health care providers. | A household contact was defined as “a person who shares the same enclosed living space for one or more nights or for frequent or extended periods during the day with the source patient during the treatment or during the three months before commencement of the current treatment”. |
| Gupta et al [45] | Botswana, Brazil, Haiti, India, Kenya, Peru, South  Africa, and Thailand | Cross-sectional | Oct 2015 to April 2016 | We assessed multidrug-resistant tuberculosis (MDR-TB) cases and their household contacts (HHCs) to inform the development of an interventional clinical trial. | A household contact was defined as a person who shared a dwelling unit and housekeeping arrangement with the index case, and who reported exposure within 6 months prior to the index case starting MDR-TB treatment. |
| Kyaw et al [24] | Myanmar | Retrospective | Apr 2016 to March 2017 | In 2016, a community-based screening of household contacts of MDR-TB patients was implemented in 33 townships in Myanmar. We assessed the implementation of this intervention, how well the screening algorithm was followed, and the yield of active TB. | A household contact is defined as “a person who shares the same enclosed living space for one or more nights or for frequent or extended periods during the day with the index case during the treatment or during the three months before the commencement of the current treatment”. |
| Malik et al [46] | Pakistan | Prospective | Feb 2016 to March 2017 | To assess the feasibility and safety of delivering treatment for presumed DR-TB infection (DR-TB preventive therapy) in a programmatic context | All individuals living in the patient’s household, defined as those sleeping under the same roof. |
| Paryani et al [47] | India | Retrospective | Index cases enrolled Jan 2016 to June 2018 | To add to the evidence around longitudinal contact tracing in addition to baseline contact tracing among drug-resistant TB cases, the present study aimed to determine the yield of systematic longitudinal household contact tracing among patients with pre-XDR-TB and XDR-TB initiated on treatment at Médecins Sans Frontières (MSF) Clinic, Mumbai. | Household contacts of DR-TB cases were members of the household regularly living with the patients registered for care in the MSF Clinic |
| Shadrach et al [48] | India | Prospective | Feb 2016 to Jan 2018 | Our study was undertaken to estimate the prevalence of MDR-TB among household contacts of MDRTB cases and gain insight into the plausible factors responsible for the spread of the disease. | Household contacts were defined as individuals who shared the same kitchen and sleeping area as the index case for at least 3 months before the diagnosis of the index case. |
| Van de Water et al [49] | South Africa | Retrospective | Index cases identified Oct 2014 to Oct 2018 | To contribute to ongoing national and provincial efforts to improve TB care in South Africa, we sought to apply the Zero TB Initiative indicators to assess prevention cascade outcomes among close contacts of DR-TB patients. | Close contacts (no definition reported) |
| Chang et al [50] | Australia | Retrospective | Index cases identified between 2000 and 2016 | The investigators sought to determine the prevalence of LTBI and active TB disease among contacts of patients with multidrug resistant (MDR)-TB in New South Wales, Australia. | The sample of MDR-TB contacts examined by this study included all individuals identified through chest clinic contact tracing programmes and comprised the population of identifiable MDR-TB contacts. |
| Kim et al [51] | Botswana  Haiti  India  Peru  South Africa  Thailand | Cross-sectional | 2015 | We characterized TB infection and TB disease in these children reported to have been exposed to RR-TB in the household. | Child (<15 years) household contacts (HHC). HHCs lived in the same dwelling unit and shared housekeeping arrangements with the index participant and reported exposure within six months prior to the index participant starting RR-TB treatment. |
| Ahmed et al [52] | Pakistan | Prospective | May 2016 to Dec 2019 | There is therefore an urgent need for more evidence on transmission in household settings to motivate the implementation of existing policies. As a part of this contact tracing study, we designed and implemented operational procedures for screening household contacts of MDR/RR-TB patients. | Household contacts – no specific definition reported |
| Ahmed et al [53] | Pakistan | Cross-sectional | Jan to Aug 2021 | Aimed to assess and address the prevalence of MDR and DS-TB among household contacts | The study population of this designs research was the close household contacts of the multiple drug resistance and Drug sensitive tuberculosis patients |
| Apolisi et al [54] | South Africa | Prospective | March 2020 to July 2021 | Médecins Sans Frontières ([MSF] Doctors without Borders), in collaboration with the Western Cape Provincial department of health and the City of Cape Town, implemented an MDR/RR-TB postexposure management program aimed at children and adolescents with household MDR/RR-TB exposure in Khayelitsha, Cape Town. This study describes the program. | Household contacts (individuals <18 years) of MDR/RR-TB index cases (no definition for household contact reported) |
| Rekart et al [55] | Tajikistan | Retrospective | Nov 2017 to Dec 2021 | In this study, we sought to evaluate household contact tracing in Dushanbe, focusing on the yield, rate of progression following exposure and risk factors.  associated with a TB diagnosis. | All household contacts (and/or their parents/guardians) were asked about TB signs/symptoms at each visit. |
