# Supplementary material for: Drug-Resistant Tuberculosis Case-Finding Strategies: Scoping Review
Source: JMIR Public Health Surveill. 2024 Jun 26;10:e46137. doi: 10.2196/46137 (PMC11237795; doi:10.2196/46137)
Supplement: Multimedia Appendix 4 [file publichealth_v10i1e46137_app4.doc]

## Wrong intervention/concept (n=45):

Abascal 2020 [1]

Abate 2012 [2]

Abubakar 2018 [3]

Aerts 2006 [4]

Alemayehu 2014 [5,6]

Allen 2021 [7]

Bohlbro 2021 [8]

Banu 2010 [9]

Barmankulova 2015 [10]

Barry 1986 [11]

Bates 2013 [12]

Bates 2013 [13]

Bates 2012 [14]

Biadglegne 2014 [15]

Buangoen 2018 [16]

Cain 2010 [17]

Cazabon 2020 [18]

CDC 2005 [19]

Cheng 2017 [20]

Cowan 2013 [21]

Dierberg 2016 [22]

Floridia 2017 [23]

Fox 2017 [24]

Freier 2006 [25]

Geerdes-Fenge 2011 [26]

Ghaderi 2020 [27]

Gurjav 2015 [28]

Gürsoy 2016 [29]

Habeenzu 2007 [30]

Han 2021 [31]

Helbling 2018 [32]

Huang 2020 [33]

Kayomo 2018 [34]

Lei 2015 [35]

Manalo 1990 [36]

Medrano 2014 [37]

Migliori 2002 [38]

Nel [6]

Oga-Omenka 2020 [39]

O’Grady 2012 [40]

Shenoi 2013 [41]

Shenoi 2017 [42]

Wang 2003 [43]

Williams 2013 [44]

Wu 2019 [45]

## Conference abstracts (n=26):

Chang 2023 [46]

Desilva 2018 [47]

Durmus 2019 [48]

Fair 2012 [49]

Fox 2012 [50]

Gebrecherkos 2019 [51]

Gupta 2020 [52]

Ha 2013 [53]

Helbling 2019 [54]

Khokhlova 2018 [55]

Kim 2020 [56]

Kimaro 2018 [57]

Lapinel 2018 [58]

Lilburn 2019 [59]

Llacer 2010 [60]

Menberu 2015 [61]

Murwira 2013 [62]

Olivianto 2012 [63]

Pankhania 2009 [64]

Paul 2014 [65]

Pepe 2018 [66]

Putri 2020 [67]

Tan 2021 [68]

Tufa 2017 [69]

Udwadia 2018 [70]

Yang 2017 [71]

## Wrong population (n=24):

Aia 2016 [72]

Ananthakrishnan 2019 [73]

Albert 2007 [74]

Alipanah 2019 [75]

Boehme 2010 [76]

Bui 2019 [77]

Da Silva Garrido 2014 [78]

Dekhil 2016 [79]

Douglas-Jones 2021 [80]

Ershova 2015 [81]

Hayward 2003 [82]

Inchai 2018 [83]

Isangula 2023 [84]

Isherwood 2013 [85]

Li 2022 [86]

Marras 2003 [87]

Maynard-Smith 2021 [88]

Miller 2018 [89]

Otchere 2022 [90]

Pimkina 2015 [91]

Sanchez-Padilla 2012 [92]

Shaik 2022 [93]

Van Kampen 2015 [94]

Venske Bierhals 2021 [95]

## Study is part of one or more of the included systematic review(s) (review(s) in brackets after citation) (n=20):

Amanullah 2014 [96] (Chiang 2021)

Attamna 2009 [97] (Fox 2013)

Bayona 2003 [98] (Chiang 2021)

Becerra 2011 [99] (Shah 2014)

Becerra 2013 [100] (Shah 2014)

Conover 2001 [101] (Fox 2013)

Chemardin 2007 [102] (Abubakar 2010)

Fox 2017 [103] (Chiang 2021)

Grandjean 2011 [104] (Shah 2014 and Kodama 2017)

Huang 2010 [105] (Shah 2014)

Johnston 2012 [106] (Kodama 2017)

Kenyon 1996 [107] (Abubakar 2010)

Leung 2013 [108] (Chiang 2021)

Mazahir 2017 [109] (Chiang 2021)

Neely 2009 [110] (Shah 2014)

Perri 2011 [111] (Shah 2014)

Qadeer 2017 [112] (Chiang 2021)

Salazar-Vergara 2003 [113] (Shah 2014 and Chiang 2021)

Singla 2011 [114] (Shah 2014)

Vella 2011 [115] (Shah 2014)

## Wrong study design/publication type (n=14)

Caminero 2015 [116]

Campbell 2006 [117]

Coleman 2012 [118]

Cox 2010 [119]

Dara 2018 [120]

Ferraro 1995 [121]

Gaskell 2019 [122]

Gomes 2023 [123]

Hong-Min 2016 [124]

Hussain 2022 [125]

Jarde 2020 [126]

Pontali 2011 [127]

Van der Werf 2012 [128]

Winetsky 2012 [129]

## Wrong outcome (n=12)

Barmina 2019 [130]

Baliashvili 2021 [131]

Becerra 2019 [132]

Kigozi 2018 [133]

Lambregts-van Weezenbeek 1998 [134]

Laniado-Laborín 2014 [135]

Lu 2018 [136]

Malik 2022 [137]

Masuda 2008 [138]

MMWR 1991 [139]

Purchase 2021 [140]

Samper 1997 [141]

## No full text (n=11)

Ahmad 2023 [142]

Albert 2006 [143]

Chang (unknown date) [144]

Chikaonda 2013 [145]

Miravet Sorribes 2016 [146]

Mehta (unknown date) [147]

Nna (unknown date) [148]

Ribeiro 2015 [149]

Trauer (unknown date) [150]

Trauer (unknown date)[151]

Smith (unknown date) [152]

## Duplicate (n=10)

Anderson 2014 [153]

Bamrah 2014 [154]

Brostrom 2011 [155]

Chang 2021 [156]

Hernan Garcia 2016 [157]

Ibrahim 2012 [158]

Kim 2023 [159]

Moro 1998 [160]

Swindells 2018 [161]

Ronnaux-Baron 2015 [162]

## To translate (n=4)

Catho 2015 [163] (French)

De Vries 2005 [164] (Nederlands)

Sasaki 2015 [165] (Japanese)

Sorribes 2016 [146] (Spanish)

## Ongoing trial (n=3)

Akalu 2024 [166]

Fox 2020 [167]

Seddon 2018 [168]

# References

1. Abascal E, Herranz M, Acosta F, Agapito J, Cabibbe AM, Monteserin J, et al. Screening of inmates transferred to Spain reveals a Peruvian prison as a reservoir of persistent Mycobacterium tuberculosis MDR strains and mixed infections. Sci Rep. 2020 Feb 17;10(1):2704. PMID: 32066749. doi: 10.1038/s41598-020-59373-w.

2. Abate D, Taye B, Abseno M, Biadgilign S. Epidemiology of anti-tuberculosis drug resistance patterns and trends in tuberculosis referral hospital in Addis Ababa, Ethiopia. BMC Res Notes. 2012 Aug 28;5:462. PMID: 22929063. doi: 10.1186/1756-0500-5-462.

3. Abubakar I, Matteelli A, de Vries G, Zenner D, Cirillo DM, Lönnroth K, et al. Towards tackling tuberculosis in vulnerable groups in the European Union: the E-DETECT TB consortium. Eur Respir J. 2018 May;51(5). PMID: 29748241. doi: 10.1183/13993003.02604-2017.

4. Aerts A, Hauer B, Wanlin M, Veen J. Tuberculosis and tuberculosis control in European prisons. Int J Tuberc Lung Dis. 2006 Nov;10(11):1215-23. PMID: 17131779.

5. Alemayehu M, Gelaw B, Abate E, Wassie L, Belyhun Y, Bekele S, et al. Active tuberculosis case finding and detection of drug resistance among HIV-infected patients: A cross-sectional study in a TB endemic area, Gondar, Northwest Ethiopia. Int J Mycobacteriol. 2014 Jun;3(2):132-8. PMID: 26786335. doi: 10.1016/j.ijmyco.2014.02.004.

6. Amy Nel H-ARMEM. Effective environmental health recommendations for Multidrug-Resistant Tuberculosis (MDR-TB) in low to middle income countries: A Systematic Review. PMID: rayyan-1182408710. doi: doi:.

7. Allen R, Calderón M, Moore DAJ, Gaskell KM, Curisinche-Rojas M, López S. Feasibility of an mobile application as a tool for multidrug-resistant tuberculosis contact monitoring in Peru. Rev Peru Med Exp Salud Publica. 2021 2021-4;38(2):272-7. PMID: rayyan-1182408713. doi: doi:10.17843/rpmesp.2021.382.6236.

8. Bohlbro AS, Hvingelby VS, Rudolf F, Wejse C, Patsche CB. Active case-finding of tuberculosis in general populations and at-risk groups: a systematic review and meta-analysis. Eur Respir J. 2021 Oct;58(4). PMID: 33766950. doi: 10.1183/13993003.00090-2021.

9. Banu S, Hossain A, Uddin MK, Uddin MR, Ahmed T, Khatun R, et al. Pulmonary tuberculosis and drug resistance in Dhaka central jail, the largest prison in Bangladesh. PLoS One. 2010 May 21;5(5):e10759. PMID: 20505826. doi: 10.1371/journal.pone.0010759.

10. Barmankulova A, Higuchi M, Sarker MA, Alim MA, Hamajima N. Tuberculosis and rifampicin resistance among migrants in kyrgyzstan: detection by a new diagnostic test. Nagoya J Med Sci. 2015 Feb;77(1-2):41-9. PMID: 25797969.

11. Barry MA, Wall C, Shirley L, Bernardo J, Schwingl P, Brigandi E, et al. Tuberculosis screening in Boston's homeless shelters. Public Health Rep. 1986 Sep-Oct;101(5):487-94. PMID: 3094079.

12. Bates M, Ahmed Y, Chilukutu L, Tembo J, Cheelo B, Sinyangwe S, et al. Use of the Xpert(®) MTB/RIF assay for diagnosing pulmonary tuberculosis comorbidity and multidrug-resistant TB in obstetrics and gynaecology inpatient wards at the University Teaching Hospital, Lusaka, Zambia. Trop Med Int Health. 2013 Sep;18(9):1134-40. PMID: 23834035. doi: 10.1111/tmi.12145.

13. Bates M, O'Grady J, Maeurer M, Tembo J, Chilukutu L, Chabala C, et al. Assessment of the Xpert MTB/RIF assay for diagnosis of tuberculosis with gastric lavage aspirates in children in sub-Saharan Africa: a prospective descriptive study. Lancet Infect Dis. 2013 Jan;13(1):36-42. PMID: 23134697. doi: 10.1016/s1473-3099(12)70245-1.

14. Bates M, O'Grady J, Mwaba P, Chilukutu L, Mzyece J, Cheelo B, et al. Evaluation of the burden of unsuspected pulmonary tuberculosis and co-morbidity with non-communicable diseases in sputum producing adult inpatients. PLoS One. 2012;7(7):e40774. PMID: 22848401. doi: 10.1371/journal.pone.0040774.

15. Biadglegne F, Sack U, Rodloff AC. Multidrug-resistant tuberculosis in Ethiopia: efforts to expand diagnostic services, treatment and care. Antimicrob Resist Infect Control. 2014;3(1):31. PMID: 25685333. doi: 10.1186/2047-2994-3-31.

16. Buangoen A, Ingviya T. Characteristics and Xpert MTB/RIF assay results of prisoners with pulmonary tuberculosis, Songkhla Province, southern Thailand. Journal of the Medical Association of Thailand. 2020 April;103(4):387-95.

17. Cain KP, Nelson LJ, Cegielski JP. Global policies and practices for managing persons exposed to multidrug-resistant tuberculosis. Int J Tuberc Lung Dis. 2010 Mar;14(3):269-74. PMID: 20132616.

18. Cazabon D, Pande T, Sen P, Daftary A, Arsenault C, Bhatnagar H, et al. User experience and patient satisfaction with tuberculosis care in low- and middle-income countries: A systematic review. J Clin Tuberc Other Mycobact Dis. 2020 May;19:100154. PMID: 32140571. doi: 10.1016/j.jctube.2020.100154.

19. *CDC*. Multidrug-resistant tuberculosis in Hmong refugees resettling from Thailand into the United States, 2004-2005. MMWR Morb Mortal Wkly Rep [Internet]. 2005 Aug-5; 54(30):[741-4 pp.]. Available from: https://www.cdc.gov/mmwr/preview/mmwrhtml/mm5430a1.htm.

20. Cheng J, Zhang H, Zhao YL, Wang LX, Chen MT. Mutual Impact of Diabetes Mellitus and Tuberculosis in China. Biomed Environ Sci. 2017 May;30(5):384-9. PMID: 28549496. doi: 10.3967/bes2017.051.

21. Cowan J, Greenberg Cowan J, Barnhart S, Demamu S, Fiseha D, Graham W, et al. A qualitative assessment of challenges to tuberculosis management and prevention in Northern Ethiopia. Int J Tuberc Lung Dis. 2013 Aug;17(8):1071-5. PMID: 23735536. doi: 10.5588/ijtld.12.0240.

22. Dierberg KL, Dorjee K, Salvo F, Cronin WA, Boddy J, Cirillo D, et al. Improved Detection of Tuberculosis and Multidrug-Resistant Tuberculosis among Tibetan Refugees, India. Emerg Infect Dis. 2016 Mar;22(3):463-8. PMID: 26889728. doi: 10.3201/eid2203.140732.

23. Floridia M, Ciccacci F, Andreotti M, Hassane A, Sidumo Z, Magid NA, et al. Tuberculosis Case Finding With Combined Rapid Point-of-Care Assays (Xpert MTB/RIF and Determine TB LAM) in HIV-Positive Individuals Starting Antiretroviral Therapy in Mozambique. Clin Infect Dis. 2017 Nov 13;65(11):1878-83. PMID: 29020319. doi: 10.1093/cid/cix641.

24. Fox GJ, Schaaf HS, Mandalakas A, Chiappini E, Zumla A, Marais BJ. Preventing the spread of multidrug-resistant tuberculosis and protecting contacts of infectious cases. Clin Microbiol Infect. 2017 Mar;23(3):147-53. PMID: 27592087. doi: 10.1016/j.cmi.2016.08.024.

25. Freier G, Wright A, Nelson G, Brenner E, Mase S, Tasker S, et al. Multidrug-resistant tuberculosis in military recruits. Emerg Infect Dis. 2006 May;12(5):760-2. PMID: 16704832. doi: 10.3201/eid1205.050708.

26. Geerdes-Fenge H, Loytved G. [Tuberculosis in 22 Au-pairs in Germany and Austria - rapid diagnosis reduces risk of infection for host families]. Dtsch Med Wochenschr. 2011 Sep;136(37):1837-41. PMID: 21898274. doi: 10.1055/s-0031-1286353.

27. Ghaderi E, Moradi GH, Sharafi S, Rahmani KH, Ahmadi SH, Mohsenpour B, et al. Tuberculosis surveillance system in the islamic republic of iran: History, structures and achievements. Iranian Journal of Epidemiology. 2020;15(4):387-96.

28. Gurjav U, Burneebaatar B, Narmandakh E, Tumenbayar O, Ochirbat B, Hill-Cawthorne GA, et al. Spatiotemporal evidence for cross-border spread of MDR-TB along the Trans-Siberian Railway line. International Journal of Tuberculosis and Lung Disease. 2015 2015-11-1;19:1376-82. PMID: rayyan-1182408529. doi: doi:https://dx.doi.org/10.5588/ijtld.15.0361.

29. Gürsoy NC, Yakupoğulları Y, Tekerekoğlu MS, Otlu B. [Evaluation of the diagnostic performance of Xpert MTB/RIF test for the detection of Mycobacterium tuberculosis and rifampin resistance in clinical samples]. Mikrobiyol Bul. 2016 Apr;50(2):196-204. PMID: 27175492. doi: 10.5578/mb.21033.

30. Habeenzu C, Mitarai S, Lubasi D, Mudenda V, Kantenga T, Mwansa J, et al. Tuberculosis and multidrug resistance in Zambian prisons, 2000-2001. Int J Tuberc Lung Dis. 2007 Nov;11(11):1216-20. PMID: 17958984.

31. Han Z, Li J, Sun G, Gu K, Zhang Y, Yao H, et al. Transmission of multidrug-resistant tuberculosis in Shimen community in Shanghai, China: a molecular epidemiology study. BMC Infectious Diseases. 2021 Oct 29;21(1):1118. PMID: 34715793. doi: https://dx.doi.org/10.1186/s12879-021-06725-0.

32. Helbling P, Kröger S, Haas W, Brusin S, Cirillo DM, Groenheit R, et al. Screening of migrants for tuberculosis identifies patients with multidrug-resistant tuberculosis but is not sufficient. Clin Microbiol Infect. 2018 Aug;24(8):918-9. PMID: 29559390. doi: 10.1016/j.cmi.2018.03.015.

33. Huang CC, Becerra MC, Calderon R, Contreras C, Galea J, Grandjean L, et al. Isoniazid Preventive Therapy in Contacts of Multidrug-Resistant Tuberculosis. Am J Respir Crit Care Med. 2020 2020-10-15;202(8):1159-68. PMID: rayyan-1182408506. doi: doi:10.1164/rccm.201908-1576OC.

34. Kayomo MK, Hasker E, Aloni M, Nkuku L, Kazadi M, Kabengele T, et al. Outbreak of Tuberculosis and Multidrug-Resistant Tuberculosis, Mbuji-Mayi Central Prison, Democratic Republic of the Congo. Emerg Infect Dis. 2018 Nov;24(11):2029-35. PMID: 30334730. doi: 10.3201/eid2411.180769.

35. Lei X, Liu Q, Escobar E, Philogene J, Zhu H, Wang Y, et al. Public-private mix for tuberculosis care and control: a systematic review. Int J Infect Dis. 2015 May;34:20-32. PMID: 25722284. doi: 10.1016/j.ijid.2015.02.015.

36. Manalo F, Tan F, Sbarbaro JA, Iseman MD. Community-based short-course treatment of pulmonary tuberculosis in a developing nation. Initial report of an eight-month, largely intermittent regimen in a population with a high prevalence of drug resistance. Am Rev Respir Dis. 1990 Dec;142(6 Pt 1):1301-5. PMID: 2123613. doi: 10.1164/ajrccm/142.6_Pt_1.1301.

37. Medrano BA, Salinas G, Sanchez C, Miramontes R, Restrepo BI, Haddad MB, et al. A missed tuberculosis diagnosis resulting in hospital transmission. Infect Control Hosp Epidemiol. 2014 May;35(5):534-7. PMID: 24709722. doi: 10.1086/675833.

38. Migliori GB, Espinal M, Danilova ID, Punga VV, Grzemska M, Raviglione MC. Frequency of recurrence among MDR-tB cases 'successfully' treated with standardised short-course chemotherapy. Int J Tuberc Lung Dis. 2002 Oct;6(10):858-64. PMID: 12365571.

39. Oga-Omenka C, Tseja-Akinrin A, Sen P, Mac-Seing M, Agbaje A, Menzies D, et al. Factors influencing diagnosis and treatment initiation for multidrug-resistant/rifampicin-resistant tuberculosis in six sub-Saharan African countries: a mixed-methods systematic review. BMJ Glob Health. 2020 Jul;5(7). PMID: 32616481. doi: 10.1136/bmjgh-2019-002280.

40. O'Grady J, Bates M, Chilukutu L, Mzyece J, Cheelo B, Chilufya M, et al. Evaluation of the Xpert MTB/RIF assay at a tertiary care referral hospital in a setting where tuberculosis and HIV infection are highly endemic. Clin Infect Dis. 2012 Nov;55(9):1171-8. PMID: 22806590. doi: 10.1093/cid/cis631.

41. Shenoi SV, Brooks RP, Catterick K, Moll AP, Friedland GH. 'Cough officer' nurses in a general medical clinic successfully detect drug-susceptible and -resistant tuberculosis. Public Health Action. 2013 Mar 21;3(1):46-50. PMID: 25392815. doi: 10.5588/pha.12.0056.

42. Shenoi SV, Moll AP, Brooks RP, Kyriakides T, Andrews L, Kompala T, et al. Integrated Tuberculosis/Human Immunodeficiency Virus Community-Based Case Finding in Rural South Africa: Implications for Tuberculosis Control Efforts. Open Forum Infect Dis. 2017 Summer;4(3):ofx092. PMID: 28695145. doi: 10.1093/ofid/ofx092.

43. Wang E, McCrann C, Notha M, Mwasekaga M, Mwansa R, Binkin N, et al. Rapid assessment of tuberculosis in a large prison system--Botswana, 2002. MMWR Morb Mortal Wkly Rep [Internet]. 2003 Mar-28; 52(12):[250-2 pp.]. Available from: https://www.cdc.gov/mmwr/preview/mmwrhtml/mm5212a3.htm.

44. Williams B, Ramroop S, Shah P, Anderson L, Das S, Riddell A, et al. Management of pediatric contacts of multidrug resistant tuberculosis in the United Kingdom. Pediatr Infect Dis J. 2013 2013-8;32(8):926-7. PMID: rayyan-1182408176. doi: doi:10.1097/INF.0b013e31829157e9.

45. Wu S, Li R, Su W, Ruan Y, Chen M, Khan MS. Is knowledge retained by healthcare providers after training? A pragmatic evaluation of drug-resistant tuberculosis management in China. BMJ Open. 2019 Mar 23;9(3):e024196. PMID: 30904847. doi: 10.1136/bmjopen-2018-024196.

46. Chang V, Nguyen V, Nguyen C, Nguyen T, Marks G, Fox G. Characteristics of household contacts of drug-resistant tuberculosis in Vietnam. Respirology. 2023 2023-3;28:228. PMID: rayyan-1182408637. doi: doi:https://dx.doi.org/10.1111/resp.14460.

47. Desilva M, Moore N, Thai D, Beth Grimm M, Sabuwala N, Brueshaber M, et al., editors. An outbreak of multidrug-resistant tuberculosis, Minnesota 2016-2017. 2018 November: Open Forum Infectious Diseases.

48. Durmus SY, Tanir G, Kaman A, Teke TA, Oz FN, editors. Evaluating children, as a part of contact tracing of an adult with tuberculosis disease, a tertiary care children hospital experience. 2019: Turkish Thoracic Journal.

49. Fair E, Miller C, Cattamanchi A, Morrison J, Hopewell PC, editors. Systematic review of tuberculosis contact investigation in low and middle income countries. American Thoracic Society International Conference, ATS; 2012: American Journal of Respiratory and Critical Care Medicine.

50. Fox GJ, Barry S, Marks GB, editors. Outcomes of contact investigation for tuberculosis: A systematic review and meta-analysis. American Thoracic Society International Conference, ATS; 2012: American Journal of Respiratory and Critical Care Medicine.

51. Gebrecherkos T, Belay T, Baye G, editors. Prevalence, HIV co-infection and multi-drug resistance of smear positive pulmonary tuberculosis in prison settings of Northwest Ethiopia. 2019: Transactions of the Royal Society of Tropical Medicine and Hygiene.

52. Gupta A, Wu X, Kim S, Naini L, Hughes M, Dawson R, et al., editors. Prevalence and incidence of tuberculosis infection and disease among household contacts exposed to rifampinresistant/multidrug resistant tuberculosis (RR/MDR-TB). 23rd International AIDS Conference Virtual; 2020: Journal of the International AIDS Society.

53. Ha YP, Littman-Quinn R, Antwi C, Seropola G, Green RS, Tesfalul MA, et al., editors. A mobile health approach to tuberculosis contact tracing in resource-limited settings. 2013: Stud Health Technol Inform Journal.

54. Helbling P, Kroger S, Haas W, Brusin S, Cirillo D, Groenheit R, et al., editors. The role of entry-screening procedures in the identification of multidrug-resistant Mycobacterium tuberculosis cluster cases amongst patients arriving in Europe from the horn of Africa, 2016-17. 2019 February: International Journal of Infectious Diseases.

55. Khokhlova Y, editor. The characteristics of TB processes in children exposed to household TB. European Respiratory Society International Congress, ERS; 2018: European Respiratory Journal.

56. Kim S, Gupta A, Wu X, Hughes MD, Dawson R, Mave V, et al., editors. Predictors of tuberculosis infection in MDR-TB household Contacts >=15 years old. 2020: Topics in Antiviral Medicine Journal.

57. Kimaro GD, Mbuya AW, Kinyaha R, Kisonga RM, Ngadaya ES, Mfinanga SG, editors. Magnitude of tuberculosis and associated factors among contacts of multi-drug resistant tuberculosis patients in Tanzania. American Thoracic Society International Conference, ATS; 2018: American Journal of Respiratory and Critical Care Medicine.

58. Lapinel N, Subhani N, Lacassagne M, Ali J, editors. Clinical and public health implications and challenges of XDR-TB management. American Thoracic Society International Conference, ATS; 2018: American Journal of Respiratory and Critical Care Medicine.

59. Lilburn P, Beiglari L, Fox G, Anthony B, editors. The Treatment Cascade: The Use of Preventative Pharmacotherapy for Latent Tb Infection Following High Risk Nosocomial Exposure to Multidrug-Resistant Tb. 2019 October: Chest journal.

60. Llacer RL, Gonong JR, Balanag V, Raymond L, Nuestro B, editors. Contact tracing of mdrtb patients at the lung center of the philippines. 2010 November: Respirology journal.

61. Menberu M, Tarekegn B, Waktola E, Wassie L, Kempker RR, Blumberg HM, et al., editors. Active tuberculosis case finding and detection of drug resistance among HIV-infected patients: A cross-sectional study in a TB Endemic Area, Gondar, Northwest Ethiopia. CHEST; 2015: Chest journal.

62. Murwira Neemanyame B, editor. Prevalence of multi-drug resistant tuberculosis and associated risk factors in HIV-positive patients registered at Mpilo Opportunistic Infection clinic, Bulawayo, Zimbabwe. 2nd International Conference on Prevention and Infection Control, ICPIC; 2013: Antimicrobial Resistance and Infection Control.

63. Olivianto E, Setyorini E, Ratnasari V, Wul, ari D, Ch, et al., editors. High infection rate of tuberculosis infection in children exposed to household contact of multidrug resistant tuberculosis. 2012 June: Paediatric Respiratory Reviews.

64. Pankhania B, Thompson C, Nash K, Marshall B, Cotterill S, Faust S, editors. Tuberculosis contact tracing at a school in a low incidence area. 2009 December: Thorax journal.

65. Paul D, Kollikkara S, editors. Spread of tuberculosis among household contacts of multi drug resistant tuberculosis (MDRTB) patients. European Respiratory Society Annual Congress; 2014: European Respiratory Journal.

66. Pepe DE, Aniskiewicz M, Paci G, Sullivan L, Dembry LM, Martinello R, et al., editors. A risk-stratifed approach to healthcare-associated tuberculosis exposures following the "stone in the pond" principle. 2018 November: Open Forum Infectious Diseases.

67. Putri C, Yulita AG, Am, a NF, Hafizmatta R, ayani D, et al., editors. High incidence of active tuberculosis among pediatric household contacts of tuberculosis patients without prior prophylactic treatment. American Thoracic Society International Conference, ATS; 2020: American Journal of Respiratory and Critical Care Medicine.

68. Tan S, Wang YT, She HW, Kwan KEL, Gan SH, Chua APG, et al. Long-term pass holders with multidrugresistant tuberculosis (MDR-TB) in Singapore: Missed opportunities for earlier diagnosis. Respirology. 2021 2021-11;26:246. PMID: rayyan-1182408227. doi: doi:https://dx.doi.org/10.1111/resp.14150.

69. Tufa TB, Nordmann T, Bosselmann M, Schonfeld A, Fuchs A, Feldt T, et al., editors. Detecting TB cases among household contacts of patients with pulmonary tb through active contact tracing in The Arsi Zone, Ethiopia. 2017 September: Open Forum Infectious Diseases.

70. Udwadia Z, Ganatra S, Poonawala H, Kishore G, Mullerpattan J, editors. Knowledge and attitudes of chest physicians regarding screening of household contacts of MDR TB patients & infection control. European Respiratory Society International Congress, ERS; 2018: European Respiratory Journal.

71. Yang SL, Wang JS, Jou R, editors. MONITOR: A novel mobile-device compatible information system for strengthening tuberculosis control. 2017 November: International Journal of Antimicrobial Agents.

72. Aia P, Kal M, Lavu E, John LN, Johnson K, Coulter C, et al. The Burden of Drug-Resistant Tuberculosis in Papua New Guinea: Results of a Large Population-Based Survey. PLoS One. 2016;11(3):e0149806. PMID: 27003160. doi: 10.1371/journal.pone.0149806.

73. Ananthakrishnan R, Richardson MD, van den Hof S, Rangaswamy R, Thiagesan R, Auguesteen S, et al. Successfully Engaging Private Providers to Improve Diagnosis, Notification, and Treatment of TB and Drug-Resistant TB: The EQUIP Public-Private Model in Chennai, India. Glob Health Sci Pract. 2019 Mar 22;7(1):41-53. PMID: 30926737. doi: 10.9745/ghsp-d-18-00318.

74. Albert H, Trollip AP, Seaman T, Abrahams C, Mole RJ, Jordaan A, et al. Evaluation of a rapid screening test for rifampicin resistance in re-treatment tuberculosis patients in the Eastern Cape. S Afr Med J. 2007 Sep;97(9):858-63. PMID: 17985057.

75. Alipanah N, Shete PB, Nguyen H, Nguyen NV, Luu L, Pham T, et al. Feasibility of Direct Sputum Molecular Testing for Drug Resistance as Part of Tuberculosis Clinical Trials Eligibility Screening. Diagnostics (Basel). 2019 May 30;9(2). PMID: 31151255. doi: 10.3390/diagnostics9020056.

76. Boehme CC, Nabeta P, Hillemann D, Nicol MP, Shenai S, Krapp F, et al. Rapid molecular detection of tuberculosis and rifampin resistance. N Engl J Med. 2010 Sep 9;363(11):1005-15. PMID: 20825313. doi: 10.1056/NEJMoa0907847.

77. Bui DP, Oren E, Roe DJ, Brown HE, Harris RB, Knight GM, et al. A Case-Control Study to Identify Community Venues Associated with Genetically-clustered, Multidrug-resistant Tuberculosis Disease in Lima, Peru. Clin Infect Dis. 2019 2019-4-24;68(9):1547-55. PMID: rayyan-1182408659. doi: doi:10.1093/cid/ciy746.

78. da Silva Garrido M, Ramasawmy R, Perez-Porcuna TM, Zaranza E, Chrusciak Talhari A, Martinez-Espinosa FE, et al. Primary drug resistance among pulmonary treatment-naïve tuberculosis patients in Amazonas State, Brazil. Int J Tuberc Lung Dis. 2014 2014-5;18(5):559-63. PMID: rayyan-1182408604. doi: doi:10.5588/ijtld.13.0191.

79. Dekhil N, Meftahi N, Mhenni B, Ben Fraj S, Haltiti R, Belhaj S, et al. MDR-TB Outbreak among HIV-Negative Tunisian Patients followed during 11 Years. PLoS One. 2016;11(4):e0153983. PMID: 27124599. doi: 10.1371/journal.pone.0153983.

80. Douglas-Jones B, Mohr-Holland E, Mema N, Mathee S, Mathews G, Hurribance S, et al. A home-based care programme for rifampicin-resistant TB. Int J Tuberc Lung Dis. 2021 Jul 1;25(7):587-9. PMID: 34183106. doi: 10.5588/ijtld.21.0051.

81. Ershova JV, Volchenkov GV, Kaminski DA, Somova TR, Kuznetsova TA, Kaunetis NV, et al. Epidemiology of Primary Multidrug-Resistant Tuberculosis, Vladimir Region, Russia. Emerg Infect Dis. 2015 Nov;21(11):2048-51. PMID: 26488585. doi: 10.3201/eid2111.150813.

82. Hayward AC, Darton T, Van-Tam JN, Watson JM, Coker R, Schwoebel V. Epidemiology and control of tuberculosis in Western European cities. Int J Tuberc Lung Dis. 2003 Aug;7(8):751-7. PMID: 12921151.

83. Inchai J, Liwsrisakun C, Bumroongkit C, Euathrongchit J, Tajarernmuang P, Pothirat C. Tuberculosis among Healthcare Workers at Chiang Mai University Hospital, Thailand: Clinical and Microbiological Characteristics and Treatment Outcomes. Jpn J Infect Dis. 2018 May 24;71(3):214-9. PMID: 29709976. doi: 10.7883/yoken.JJID.2017.274.

84. Isangula K, Philbert D, Ngari F, Ajeme T, Kimaro G, Yimer G, et al. Implementation of evidence-based multiple focus integrated intensified TB screening to end TB (EXIT-TB) package in East Africa: a qualitative study. BMC Infectious Diseases. 2023 2023-12;23(161). PMID: rayyan-1182408498. doi: doi:https://dx.doi.org/10.1186/s12879-023-08069-3.

85. Isherwood LE, editor. 'The evaluation of rapid screening of M/XDR-TB patients within a dedicated M/XDR-TB hospital in Gauteng, South Africa'. 2013.

86. Li M, Guo M, Peng Y, Jiang Q, Xia L, Zhong S, et al. High proportion of tuberculosis transmission among social contacts in rural China: a 12-year prospective population-based genomic epidemiological study. Emerg Microbes Infect. 2022 2022-12;11(1):2102-11. PMID: rayyan-1182408431. doi: doi:10.1080/22221751.2022.2112912.

87. Marras TK, Wilson J, Wang EE, Avendano M, Yang JW. Tuberculosis among Tibetan refugee claimants in Toronto: 1998 to 2000. Chest. 2003 Sep;124(3):915-21. PMID: 12970017. doi: 10.1378/chest.124.3.915.

88. Maynard-Smith L, Brown CS, Harris RJ, Hodkinson P, Tamne S, Anderson SR, et al. Contact tracing following in-flight exposure to TB: Why the 8-hour rule? International Journal of Tuberculosis and Lung Disease. 2021 2021-7-1;25:593-5. PMID: rayyan-1182408397. doi: doi:https://dx.doi.org/10.5588/ijtld.21.0141.

89. Miller AC, Livchits V, Ahmad Khan F, Atwood S, Kornienko S, Kononenko Y, et al. Turning Off the Tap: Using the FAST Approach to Stop the Spread of Drug-Resistant Tuberculosis in the Russian Federation. J Infect Dis. 2018 Jul 13;218(4):654-8. PMID: 29659912. doi: 10.1093/infdis/jiy190.

90. Otchere ID, Morgan PA, Asare P, Osei-Wusu S, Aboagye SY, Yirenkyi SO, et al. Analysis of drug resistance among difficult-to-treat tuberculosis patients in Ghana identifies several pre-XDR TB cases. Front Microbiol. 2022 2022;13:1069292. PMID: rayyan-1182408339. doi: doi:10.3389/fmicb.2022.1069292.

91. Pimkina E, Zablockis R, Nikolayevskyy V, Danila E, Davidaviciene E. The Xpert® MTB/RIF assay in routine diagnosis of pulmonary tuberculosis: A multicentre study in Lithuania. Respir Med. 2015 Nov;109(11):1484-9. PMID: 26403251. doi: 10.1016/j.rmed.2015.07.006.

92. Sanchez-Padilla E, Dlamini T, Ascorra A, Rüsch-Gerdes S, Tefera ZD, Calain P, et al. High prevalence of multidrug-resistant tuberculosis, Swaziland, 2009-2010. Emerg Infect Dis. 2012 Jan;18(1):29-37. PMID: 22260950. doi: 10.3201/eid1801.110850.

93. Shaik J, Pillay M, Moodley J, Jeena P. Predominance of the Mycobacterium tuberculosis Beijing strain amongst children from a high tuberculosis burden township in South Africa. Tuberculosis. 2022 2022-9;136(102250). PMID: rayyan-1182408271. doi: doi:https://dx.doi.org/10.1016/j.tube.2022.102250.

94. van Kampen SC, Tursynbayeva A, Koptleuova A, Murzakhmetova Z, Bigalieva L, Aubakirova M, et al. Effect of Introducing Xpert MTB/RIF to Test and Treat Individuals at Risk of Multidrug-Resistant Tuberculosis in Kazakhstan: A Prospective Cohort Study. PLoS One. 2015;10(7):e0132514. PMID: 26181578. doi: 10.1371/journal.pone.0132514.

95. Venske Bierhals D, Busatto C, Silveira M, da Matta Talaier E, Silva ABS, Reis AJ, et al. Tuberculosis cases in a prison in the extreme south of Brazil. J Med Microbiol. 2021 Mar;70(3). PMID: 33555247. doi: 10.1099/jmm.0.001319.

96. Amanullah F, Ashfaq M, Khowaja S, Parekh A, Salahuddin N, Lotia-Farrukh I, et al. High tuberculosis prevalence in children exposed at home to drug-resistant tuberculosis. Int J Tuberc Lung Dis. 2014 May;18(5):520-7. PMID: 24903786. doi: 10.5588/ijtld.13.0593.

97. Attamna A, Chemtob D, Attamna S, Fraser A, Rorman E, Paul M, et al. Risk of tuberculosis in close contacts of patients with multidrug resistant tuberculosis: a nationwide cohort. Thorax. 2009 2009-3;64(3):271. PMID: rayyan-1182408694. doi: doi:10.1136/thx.2008.100974.

98. Bayona J, Chavez-Pachas AM, Palacios E, Llaro K, Sapag R, Becerra MC. Contact investigations as a means of detection and timely treatment of persons with infectious multidrug-resistant tuberculosis. Int J Tuberc Lung Dis. 2003 Dec;7(12 Suppl 3):S501-9. PMID: 14677844.

99. Becerra MC, Appleton SC, Franke MF, Chalco K, Arteaga F, Bayona J, et al. Tuberculosis burden in households of patients with multidrug-resistant and extensively drug-resistant tuberculosis: a retrospective cohort study. Lancet. 2011 Jan 8;377(9760):147-52. PMID: 21145581. doi: 10.1016/s0140-6736(10)61972-1.

100. Becerra MC, Franke MF, Appleton SC, Joseph JK, Bayona J, Atwood SS, et al. Tuberculosis in children exposed at home to multidrug-resistant tuberculosis. Pediatr Infect Dis J. 2013 Feb;32(2):115-9. PMID: 22926210. doi: 10.1097/INF.0b013e31826f6063.

101. Conover C, Ridzon R, Valway S, Schoenstadt L, McAuley J, Onorato I, et al. Outbreak of multidrug-resistant tuberculosis at a methadone treatment program. Int J Tuberc Lung Dis. 2001 2001-1;5(1):59-64. PMID: rayyan-1182408612. doi: doi:.

102. Chemardin J, Paty MC, Renard-Dubois S, Veziris N, Antoine D. Contact tracing of passengers exposed to an extensively drug-resistant tuberculosis case during an air flight from Beirut to Paris, October 2006. Euro Surveill. 2007 Dec 6;12(12):E071206.2. PMID: 18067848. doi: 10.2807/esw.12.49.03325-en.

103. Fox GJ, Anh NT, Nhung NV, Loi NT, Hoa NB, Ngoc Anh LT, et al. Latent tuberculous infection in household contacts of multidrug-resistant and newly diagnosed tuberculosis. Int J Tuberc Lung Dis. 2017 Mar 1;21(3):297-302. PMID: 28225339. doi: 10.5588/ijtld.16.0576.

104. Grandjean L, Crossa A, Gilman RH, Herrera C, Bonilla C, Jave O, et al. Tuberculosis in household contacts of multidrug-resistant tuberculosis patients. Int J Tuberc Lung Dis. 2011 Sep;15(9):1164-9, i. PMID: 21943839. doi: 10.5588/ijtld.11.0030.

105. Huang YW, Shen GH, Lee JJ, Yang WT. Latent tuberculosis infection among close contacts of multidrug-resistant tuberculosis patients in central Taiwan. Int J Tuberc Lung Dis. 2010 2010-11;14(11):1430-5. PMID: rayyan-1182408505. doi: doi:.

106. Johnston J, Admon A, Ibrahim A, Elwood K, Tang P, Cook V, et al. Long term follow-up of drug resistant and drug susceptible tuberculosis contacts in a Low incidence setting. BMC Infect Dis. 2012 Oct 22;12:266. PMID: 23088397. doi: 10.1186/1471-2334-12-266.

107. Kenyon TA, Valway SE, Ihle WW, Onorato IM, Castro KG. Transmission of multidrug-resistant Mycobacterium tuberculosis during a long airplane flight. N Engl J Med. 1996 1996-4-11;334(15):933-8. PMID: rayyan-1182408464. doi: doi:10.1056/nejm199604113341501.

108. Leung EC, Leung CC, Kam KM, Yew WW, Chang KC, Leung WM, et al. Transmission of multidrug-resistant and extensively drug-resistant tuberculosis in a metropolitan city. Eur Respir J. 2013 Apr;41(4):901-8. PMID: 22878878. doi: 10.1183/09031936.00071212.

109. Mazahir R, Beig FK, Ahmed Z, Alam S. Burden of tuberculosis among household children of adult multi drug resistant patients and their response to first line anti tubercular drugs. Egyptian Pediatric Association Gazette. 2017 2017/11/01/;65(4):122-6. doi: https://doi.org/10.1016/j.epag.2017.09.002.

110. Neely F, Maguire H, Le Brun F, Davies A, Gelb D, Yates S. High rate of transmission among contacts in large London outbreak of isoniazid mono-resistant tuberculosis. J Public Health (Oxf). 2010 Mar;32(1):44-51. PMID: 19542269. doi: 10.1093/pubmed/fdp056.

111. Perri BR, Proops D, Moonan PK, Munsiff SS, Kreiswirth BN, Kurepina N, et al. Mycobacterium tuberculosis cluster with developing drug resistance, New York, New York, USA, 2003-2009. Emerg Infect Dis. 2011 Mar;17(3):372-8. PMID: 21392426. doi: 10.3201/eid1703.101002.

112. Qadeer E, Fatima R, Haq MU, Yaqoob A, Kyaw NTT, Shah S, et al. Yield of facility-based verbal screening amongst household contacts of patients with multi-drug resistant tuberculosis in Pakistan. J Clin Tuberc Other Mycobact Dis. 2017 May;7:22-7. PMID: 31723697. doi: 10.1016/j.jctube.2017.01.004.

113. Salazar-Vergara RM, Sia IG, Tupasi TE, Alcañeses MR, Orillaza RB, Co V, et al. Tuberculosis infection and disease in children living in households of Filipino patients with tuberculosis: a preliminary report. Int J Tuberc Lung Dis. 2003 Dec;7(12 Suppl 3):S494-500. PMID: 14677843.

114. Singla N, Singla R, Jain G, Habib L, Behera D. Tuberculosis among household contacts of multidrug-resistant tuberculosis patients in Delhi, India. Int J Tuberc Lung Dis. 2011 Oct;15(10):1326-30. PMID: 22283889. doi: 10.5588/ijtld.10.0564.

115. Vella V, Racalbuto V, Guerra R, Marra C, Moll A, Mhlanga Z, et al. Household contact investigation of multidrug-resistant and extensively drug-resistant tuberculosis in a high HIV prevalence setting. Int J Tuberc Lung Dis. 2011 Sep;15(9):1170-5, i. PMID: 21943840. doi: 10.5588/ijtld.10.0781.

116. Caminero JA, Migliori GB. Automated Digital Microscopy in New Tuberculosis Diagnostic Algorithms. Can It Boost Case Finding? Am J Respir Crit Care Med. 2015 Jun 15;191(12):1352-3. PMID: 26075421. doi: 10.1164/rccm.201504-0790ED.

117. Campbell IA, Bah-Sow O. Pulmonary tuberculosis: diagnosis and treatment. Bmj. 2006 May 20;332(7551):1194-7. PMID: 16709993. doi: 10.1136/bmj.332.7551.1194.

118. Coleman CH, Selgelid MJ, Reis A, Reichman LB, Jaramillo E. The role of informed consent in tuberculosis testing and screening. Eur Respir J. England2012. p. 1057-9.

119. Cox H, van Cutsem G. Household screening and multidrug-resistant tuberculosis. Lancet. 2011 Jan 8;377(9760):103-4. PMID: 21145582. doi: 10.1016/s0140-6736(10)61390-6.

120. Dara M, Zachariah R. Ending tuberculosis calls for leaving no one behind. Lancet Infect Dis. 2018 Apr;18(4):365-6. PMID: 29326014. doi: 10.1016/s1473-3099(17)30746-6.

121. Ferraro MJ, Klietmann W, Lee JT. Diagnosis and detection of drug-resistant strains of M. tuberculosis. AIDS Clin Care. 1995 Apr;7(4):27-9, 36. PMID: 11370656.

122. Gaskell KM, Allen R, Moore DAJ. Exposed! Management of MDR-TB household contacts in an evidence light era. Int J Infect Dis. 2019 2019-3;80:S13-s6. PMID: rayyan-1182408554. doi: doi:10.1016/j.ijid.2019.02.037.

123. Gomes I, Garg T, Churchyard G, Gupta A, Hesseling AC, Swindells S, et al. The cascade of care for household contacts of people with drug-resistant TB. Int J Tuberc Lung Dis. 2023 2023-2-1;27(2):154-6. PMID: rayyan-1182408548. doi: doi:10.5588/ijtld.22.0473.

124. Hong-Min W, Xiao-Hong Z, Jing W. Drug-resistant Tuberculosis and the Prevention of Ongoing Transmission. Clin Infect Dis. 2016 2016-1-15;62(2):266-7. PMID: rayyan-1182408508. doi: doi:10.1093/cid/civ832.

125. Hussain H, Malik AA. Investing in drug-resistant tuberculosis household contact management and preventive treatment. The Lancet Global Health. 2022 2022-7;10:e942-e3. PMID: rayyan-1182408502. doi: doi:https://dx.doi.org/10.1016/S2214-109X%2822%2900200-5.

126. A meta-review of systematic reviews of chronic disease multimorbidity in people with tuberculosis in low- and middle-income countries [database on the Internet]. 2020. Available from: https://www.crd.york.ac.uk/prospero/display_record.php?RecordID=209012.

127. Pontali E, Sotgiu G, Migliori GB. Management of MDR-TB household contacts: how difficult is it to climb the mountain? Int J Tuberc Lung Dis. 2011 Sep;15(9):1137-8. PMID: 21943835. doi: 10.5588/ijtld.11.0225.

128. van der Werf MJ, Sandgren A, Manissero D. Management of contacts of multidrug-resistant tuberculosis patients in the European Union and European Economic Area. Int J Tuberc Lung Dis. 2012;16(3):426. PMID: 22640458. doi: 10.5588/ijtld.11.0605.

129. Winetsky DE, Negoescu DM, DeMarchis EH, Almukhamedova O, Dooronbekova A, Pulatov D, et al. Screening and rapid molecular diagnosis of tuberculosis in prisons in Russia and Eastern Europe: a cost-effectiveness analysis. PLoS Med. 2012;9(11):e1001348. PMID: 23209384. doi: 10.1371/journal.pmed.1001348.

130. Barmina NA, Baryshnikova LA. Manifestations of tuberculosis infection in children and adolescents who had confirmed contacts with tuberculosis patients on the example of Perm region. [Russian]. Voprosy Prakticheskoi Pediatrii. 2019;14(2):21-8.

131. Baliashvili D, Gandhi NR, Kim S, Hughes M, Mave V, Mendoza-Ticona A, et al. Resistance to Mycobacterium tuberculosis Infection Among Household Contacts: A Multinational Study. Clinical Infectious Diseases. 2021 2021;73(6):1037-45. PMID: rayyan-1182408685. doi: doi:10.1093/cid/ciab269.

132. Becerra MC, Huang CC, Lecca L, Bayona J, Contreras C, Calderon R, et al. Transmissibility and potential for disease progression of drug resistant Mycobacterium tuberculosis: prospective cohort study. Bmj. 2019 2019-10-24;367:l5894. PMID: rayyan-1182408676. doi: doi:10.1136/bmj.l5894.

133. Kigozi G, Engelbrecht M, Heunis C, Janse van Rensburg A. Household contact non-attendance of clinical evaluation for tuberculosis: a pilot study in a high burden district in South Africa. BMC Infect Dis. 2018 Mar 5;18(1):106. PMID: 29506488. doi: 10.1186/s12879-018-3010-3.

134. Lambregts-van Weezenbeek CS, Jansen HM, Veen J, Nagelkerke NJ, Sebek MM, van Soolingen D. Origin and management of primary and acquired drug-resistant tuberculosis in The Netherlands: the truth behind the rates. Int J Tuberc Lung Dis. 1998 Apr;2(4):296-302. PMID: 9559400.

135. Laniado-Laborín R, Cazares-Adame R, Volker-Soberanes ML, del Portillo-Mustieles C, Villa-Rosas C, Oceguera-Palao L, et al. Latent tuberculous infection prevalence among paediatric contacts of drug-resistant and drug-susceptible cases. Int J Tuberc Lung Dis. 2014 2014-5;18(5):515-9. PMID: rayyan-1182408441. doi: doi:10.5588/ijtld.13.0840.

136. Lu P, Ding X, Liu Q, Lu W, Martinez L, Sun J, et al. Mediating Effect of Repeated Tuberculosis Exposure on the Risk of Transmission to Household Contacts of Multidrug-Resistant Tuberculosis Patients. Am J Trop Med Hyg. 2018 2018-2;98(2):364-71. PMID: rayyan-1182408420. doi: doi:10.4269/ajtmh.17-0325.

137. Malik AA, Siddique M, Chandir S, Jaswal M, Siddiqui S, Fuad J, et al. Travel reimbursements, distance to health facility and preventive treatment cascade for drug-resistant TB. International Journal of Tuberculosis and Lung Disease. 2022 2022-8-1;26:789-91. PMID: rayyan-1182408406. doi: doi:https://dx.doi.org/10.5588/ijtld.22.0204.

138. Masuda M, Harada N, Shishido S, Higuchi K, Mori T. [Usefulness of QuantiFERONTB-2G in a suspected case of drug resistant tuberculosis outbreak in a university]. Kekkaku. 2008 Jan;83(1):7-11. PMID: 18283909.

139. Transmission of multidrug-resistant tuberculosis from an HIV-positive client in a residential substance-abuse treatment facility--Michigan. MMWR Morb Mortal Wkly Rep. 1991 1991-3-1;40(8):129-31. PMID: rayyan-1182408762. doi: doi:.

140. Purchase S, Batist E, Mmile N, Nkosi S, Workman J, Martinson N, et al. Challenges in recruiting children to a multidrug-resistant TB prevention trial. International Journal of Tuberculosis & Lung Disease. 2021 10 01;25(10):814-22. PMID: 34615578. doi: https://dx.doi.org/10.5588/ijtld.21.0098.

141. Samper S, Martín C, Pinedo A, Rivero A, Blázquez J, Baquero F, et al. Transmission between HIV-infected patients of multidrug-resistant tuberculosis caused by Mycobacterium bovis. Aids. 1997 1997-8;11(10):1237-42. PMID: rayyan-1182408287. doi: doi:10.1097/00002030-199710000-00006.

142. Ahmad S, Iqbal J, Zafar S, Aslam J, Zahid H, Rahman WU. Efficacy of GeneXpert in Active Case Detection Among Contacts of Drug Resistant TB Patients. Medical Forum Monthly. 2023 2023-4;34:80-4. PMID: rayyan-1182408720. doi: doi:.

143. Albert H. A cost-effectiveness analysis of drug resistance screening in the management of tuberculosis in South Africa. 2006.

144. Chang W, Ling R, Chiam D, Britton W, Fox G. Characterising the treatment and prevention of drug resistant tuberculosis in NSW. Respirology. March;23:134.

145. Chikaonda T. Evaluation of the xpert MTB/RIT assay and its impact on tuberculosis diagnosis and rifampicin resistance screening: efforts to determine the prevalence of drug resistant TB and rpo B Gene mutations in the Malawian adult population. 2013.

146. Miravet Sorribes L, Arnedo Pena A, Bellido Blasco JB, Romeu García MA, Gil Fortuño M, García Sidro P, et al. Outbreak of multidrug-resistant tuberculosis in two secondary schools. Arch Bronconeumol. 2016 Feb;52(2):70-5. PMID: 25987369. doi: 10.1016/j.arbres.2015.03.010.

147. Mehta J, Keith R, Al Hasan M, Ryl, B, Roy T. Mini epidemic of isoniazide resistant TB in rural TN: a need for supervised preventive therapy. Tenn Med. Aug;102(8):41-4.

148. Nna E, Obu D, Garba M, Uzoma B, Chinaza E, Khalid R, et al. Multidrug-resistant tuberculosis in Africa: a protocol for systematic review and meta-analysis.

149. Ribeiro I, Dlamini S, Avenant T, Du Plessis N. Contact tracing and management of children and adolescents in a place of safety after drug-resistant tuberculosis exposure. Southern African Journal of Epidemiology and Infection. 2015;30:171-2.

150. Trauer J, Denholm J, McBryde E, Majumdar S. Is multi-drug resistant tuberculosis more or less transmissible than drug-susceptible tuberculosis? A systematic review.

151. Trauer J, Doan T, Scott N, Denholm J, McBryde E. Allocative efficiency in tuberculosis control.

152. Smith J, Peterson S, Gandhi N, Cohen T, Dowdy D, Strestha S. Differing genetic techniques in identifying tuberculosis transmission clusters and their impact on transmission inference: a systematic review.

153. Anderson LF, Tamne S, Brown T, Watson JP, Mullarkey C, Zenner D, et al. Transmission of multidrug-resistant tuberculosis in the UK: a cross-sectional molecular and epidemiological study of clustering and contact tracing. Lancet Infect Dis. 2014 May;14(5):406-15. PMID: 24602842. doi: 10.1016/s1473-3099(14)70022-2.

154. Bamrah S, Brostrom R, Dorina F, Setik L, Song R, Kawamura LM, et al. Treatment for LTBI in contacts of MDR-TB patients, Federated States of Micronesia, 2009-2012. Int J Tuberc Lung Dis. 2014 2014-8;18(8):912-8. PMID: rayyan-1182408682. doi: doi:10.5588/ijtld.13.0028.

155. Brostrom R, Fred D, Heetderks A, Desai M, Song R, Haddad M, et al. Islands of hope: building local capacity to manage an outbreak of multidrug-resistant tuberculosis in the Pacific. American Journal of Public Health. 2011 2011;101(1):14-8. PMID: rayyan-1182408663. doi: doi:10.2105/AJPH.2009.177170.

156. Chang V, Ling RH, Velen K, Fox GJ. Latent tuberculosis infection among contacts of patients with multidrug-resistant tuberculosis in New South Wales, Australia. ERJ Open Res. 2021 2021-7;7(3). PMID: rayyan-1182408638. doi: doi:10.1183/23120541.00149-2021.

157. Hernan Garcia C, Moreno Cea L, Fernandez Espinilla V, Ruiz Lopez Del Prado G, Fernandez Arribas S, Andres Garcia I, et al. Outbreak of isoniazid-resistant tuberculosis in an immigrant community in Spain. Archivos de Bronconeumologia. 2016 Jun;52(6):289-92. PMID: 26584529. doi: https://dx.doi.org/10.1016/j.arbres.2015.07.014.

158. Ibrahim A, Johnston J, Admon A, Elwood K, Tang P, Cook V, et al. Long-term follow-up of multidrug-resistant tuberculosis contacts in a low incidence setting. American Journal of Respiratory and Critical Care Medicine. 2012;185.

159. Kim S, Hesseling AC, Wu X, Hughes MD, Sarita Shah N, Gaikwad S, et al. Factors associated with prevalent Mycobacterium tuberculosis infection and disease among adolescents and adults exposed to rifampin-resistant tuberculosis in the household. PLoS ONE. 2023 2023-3;18. PMID: rayyan-1182408456. doi: doi:https://dx.doi.org/10.1371/journal.pone.0283290.

160. Moro ML, Gori A, Errante I, Infuso A, Franzetti F, Sodano L, et al. An outbreak of multidrug-resistant tuberculosis involving HIV-infected patients of two hospitals in Milan, Italy. Aids. 1998 1998-6-18;12:1095-102. PMID: rayyan-1182408373. doi: doi:http://dx.doi.org/10.1097/00002030-199809000-00018.

161. Swindells S, Gupta A, Kim S, Hughes MD, Sanchez J, Mave V, et al. Resource utilization for multidrug-resistant tuberculosis household contact investigations (A5300/I2003). Int J Tuberc Lung Dis. 2018 Sep 1;22(9):1016-22. PMID: 30092866. doi: 10.5588/ijtld.18.0163.

162. Ronnaux-Baron AS, Dumitriescu O, Ader F, Biron F, Boibieux A, Bouaziz A, et al. Children exposed to multidrug-resistant tuberculosis: How should we manage? Analysis of 46 child contacts and review of the literature. [French]. Revue de Pneumologie Clinique. 2015 01-Dec;71(6):335-41.

163. Catho G, Sénéchal A, Ronnaux-Baron AS, Valour F, Perpoint T, Bouaziz A, et al. [Children exposed to multidrug-resistant tuberculosis: How should we manage? Analysis of 46 child contacts and review of the literature]. Rev Pneumol Clin. 2015 Dec;71(6):335-41. PMID: 26195117. doi: 10.1016/j.pneumo.2015.05.003.

164. de Vries G, van Altena R, van Soolingen D, Broekmans JF, van Hest NA. [An outbreak of multiresistant tuberculosis from Eastern Europe in the Netherlands]. Ned Tijdschr Geneeskd. 2005 Aug 27;149(35):1921-4. PMID: 16159027.

165. Sasaki Y, Yamagishi F, Yagi T. [Current situation of contacts examination and chemoprophylaxis for persons exposed to multi-drug resistant tuberculosis in ordinance-designated cities in Japan]. Kekkaku. 2005 Oct;80(10):637-42. PMID: 16296391.

166. Temesgen Akalu HWKAACEGFS. The burden of multidrug-resistant tuberculosis among household contacts: a systematic review and meta-analysis. PMID: rayyan-1182408224. doi: doi:.

167. Fox GJ, Nguyen CB, Nguyen TA, Tran PT, Marais BJ, Graham SM, et al. Levofloxacin versus placebo for the treatment of latent tuberculosis among contacts of patients with multidrug-resistant tuberculosis (the VQUIN MDR trial): a protocol for a randomised controlled trial. BMJ Open. 2020 Jan 2;10(1):e033945. PMID: 31900274. doi: 10.1136/bmjopen-2019-033945.

168. Seddon JA, Garcia-Prats AJ, Purchase SE, Osman M, Demers AM, Hoddinott G, et al. Levofloxacin versus placebo for the prevention of tuberculosis disease in child contacts of multidrug-resistant tuberculosis: study protocol for a phase III cluster randomised controlled trial (TB-CHAMP). Trials. 2018 2018-12-20;19(1):693. PMID: rayyan-1182408275. doi: doi:10.1186/s13063-018-3070-0.
